# Peroxiredoxin alleviates the fitness costs of imidacloprid resistance in an insect pest of rice

**DOI:** 10.1371/journal.pbio.3001190

**Published:** 2021-04-12

**Authors:** Rui Pang, Ke Xing, Longyu Yuan, Zhikun Liang, Meng Chen, Xiangzhao Yue, Yi Dong, Yan Ling, Xionglei He, Xianchun Li, Wenqing Zhang

**Affiliations:** 1 State Key Laboratory of Biocontrol, School of Life Sciences, Sun Yat-sen University, Guangzhou, Guangdong, China; 2 Guangdong Provincial Key Laboratory of Microbial Safety and Health, State Key Laboratory of Applied Microbiology Southern China, Institute of Microbiology, Guangdong Academy of Science, Guangzhou, Guangdong, China; 3 Institute of Plant Protection, Guangxi Academy of Agricultural Sciences, Nanning, Guangxi, China; 4 Department of Entomology and BIO5 Institute, University of Arizona, Tucson, Arizona, United States of America; University of Notre Dame, UNITED STATES

## Abstract

Chemical insecticides have been heavily employed as the most effective measure for control of agricultural and medical pests, but evolution of resistance by pests threatens the sustainability of this approach. Resistance-conferring mutations sometimes impose fitness costs, which may drive subsequent evolution of compensatory modifier mutations alleviating the costs of resistance. However, how modifier mutations evolve and function to overcome the fitness cost of resistance still remains unknown. Here we show that overexpression of P450s not only confers imidacloprid resistance in the brown planthopper, *Nilaparvata lugens*, the most voracious pest of rice, but also leads to elevated production of reactive oxygen species (ROS) through metabolism of imidacloprid and host plant compounds. The inevitable production of ROS incurs a fitness cost to the pest, which drives the increase or fixation of the compensatory modifier allele T65549 within the promoter region of *N*. *lugens peroxiredoxin* (*NlPrx*) in the pest populations. T65549 allele in turn upregulates the expression of *NlPrx* and thus increases resistant individuals’ ability to clear the cost-incurring ROS of any source. The frequent involvement of P450s in insecticide resistance and their capacity to produce ROS while metabolizing their substrates suggest that peroxiredoxin or other ROS-scavenging genes may be among the common modifier genes for alleviating the fitness cost of insecticide resistance.

## Abstract 2

This study shows that peroxiredoxin is a modifier gene whose cis-upregulation in the brown planthopper (Nilaparvata lugens) increases the ability of resistant populations to clear the fitness cost-incurring reactive oxygen species that inevitably result from P450-meditated metabolism of imidacloprid insecticide.

## Introduction

Arthropod pests pose a serious threat to both agriculture and human health through ingestion of agricultural produce and transmission of plant, livestock, and human pathogens. Worldwide, these deadly pests cause approximately 20% of crop yield loss annually despite the current crop protection practice [1], and are responsible for more than 17% of infectious diseases [2]. Since the initial spectacular success of organic insecticides in the 1940s, chemical insecticides, and, more recently, transgenic crops expressing insecticidal toxins from *Bacillus thuringiensis* (Bt), have been heavily employed as the most effective measures for control of arthropod pests [3]. The overreliance on insecticides constitutes a strong selective pressure for the evolution of insecticide-resistant populations of pests. As a result, populations of more than 600 pest species had developed resistance to 1 or more insecticides up to 2019, and resistance to 335 insecticides of various chemical classes and 9 Bt toxins had already evolved in 1 or more arthropod species [4,5]. Such widespread evolution of insecticide resistance represents a major challenge to sustainable management of arthropod pests, agriculture, and public health [3,6].

Insecticide resistance is known to evolve through genetic/genomic mutations that increase behavioral avoidance of the insecticide, reduce penetration through the cuticle, decrease target site (e.g., sodium channel, acetylcholinesterase [AchE], acetylcholine receptor [AchR]) sensitivity, increase metabolism by detoxification enzymes (e.g., cytochrome P450 monooxygenases [P450s], esterases, UDP-glucosyltransferases [UGTs], and glutathione S-transferases [GSTs]), and/or enhance efflux and excretion by ATP-binding cassette (ABC) [7–9]. Elevated metabolism, the most common resistance mechanism, is achieved by gene amplification, *cis*/*trans* upregulation, conversion, and/or coding sequence point mutation of detoxification genes [7, 8,10,11]. By contrast, target site insensitivity, the second most common resistance mechanism, is acquired largely through conserved point mutations and, to a much lesser extent, small deletion, premature stop codon, and mis-splicing [8,12,13].

The aforementioned genetic/genomic mutations conferring insecticide resistance sometimes impose a large fitness cost on the pest in the absence of the insecticide [6,14,15]. This is not surprising as the resistance-conferring point mutations in the essential target enzyme/protein not only prevent or reduce insecticide binding, but also make the target enzyme suboptimal compared to its evolutionarily optimized “wild-type” susceptible allele, yielding unwanted negative pleiotropic effects on fitness. Overproduction of detoxification enzymes by gene amplification or upregulation is an energetically costly process that takes resources and energy away from the growth, development, and/or reproduction of arthropod pests [14]. Consequently, resistant insects often exhibit lower survival [16], higher overwintering mortality [17,18], lower fecundity [19,20], longer development time [20,21], shorter adult longevity, less energy reserves [22], lower tolerance to plant defense compounds [23], and larger fluctuation asymmetry [24,25] compared with their susceptible counterparts.

The fitness costs incurred by the mutated resistant allele of the target or detoxification gene may be mitigated or even eliminated by compensatory genes known as modifiers, selected by continued use of an insecticide following the emergence of resistance [6,14,15]. Such a compensatory evolution of modifier genes has been observed in the resistance of the Australian sheep blowfly (*Lucilia cuprina*) to diazinon, which was first detected in 1965, 7 years after the introduction of diazinon to control the blowfly in Australia [26]. The diazinon resistance is conferred by a mutation (G137D) that converts the E3 carboxylesterase encoded by *LcαE7* at the Rop-1 locus to an organophosphorus hydrolase, causing a fitness cost and fluctuating asymmetry initially [27,28]. Continued use of diazinon led to the disappearance of fitness costs and fluctuating asymmetry in the field populations since the late 1970s [29], because a second mutation was selected in a modifier gene mapped to the *Scalloped wings* (*Scl*) locus [24,29,30]. Such cost-free resistance mutations have been also found in *CYP6G1*-upregulation-mediated DDT resistance in *Drosophila melanogaster* [31], P450-mediated permethrin resistance in *Culex pipiens quinquefasciatus* [22], pyrethroid-resistant *Sitophilus zeamais* [32], Bt-resistant *Plutella xylostella* [33], and acetamiprid- or pyriproxyfen-resistant *Bemisia tabaci* [34,35], indicating the potential prevalence of fitness modifier genes. However, to date, other than knowing the candidate for the fitness modifier gene of diazinon resistance in the blowfly, little is known as to how modifier genes function to overcome the fitness cost of resistance mutations.

We chose the resistance of the brown planthopper (*Nilaparvata lugens*) to the neonicotinoid insecticide imidacloprid to address this key question of how modifier genes functionally mitigate the fitness costs of resistance mutations. In the early 1990s, imidacloprid was introduced to control *N*. *lugens* [36], one of the most destructive rice pests in Asia. A low level of imidacloprid resistance was first detected in 1997 in a field population from Guilin, Guangxi [37]. By 2005, moderate to high levels of imidacloprid resistance (resistance ratio ranging from 79 to 811) were widespread, resulting in field control failure in 2005 [38,39]. The resistance still remains at an extremely high level because of continued use of neonicotinoid insecticides, including imidacloprid [39]. Upregulation of *CYP6ER1* with a gain of function mutation, and occasionally upregulation of *CYP6AY1*, is responsible for imidacloprid resistance in field populations of *N*. *lugens* across Asia [40–44]. The upregulation of the 2 P450 genes comes with a significant fitness cost, as evidenced by significantly lower larval survival, adult emergence, copulation, fecundity, and hatchability in a laboratory-selected strain [45] and the rapid drop of imidacloprid resistance in both the laboratory- and field-selected populations of *N*. *lugens* after stopping selection with imidacloprid, or with reduced application of imidacloprid [36,46]. However, that the resistance never disappeared but stabilized at the level of 100- to 230-fold in the absence of imidacloprid [46] demonstrates that a fitness-improving modifier gene has already evolved in the field populations of *N*. *lugens*. In this study, we identified and confirmed that the *N*. *lugens peroxiredoxin* (*NlPrx*) gene is a modifier gene, whose *cis* upregulation increases resistant populations’ ability to clear the fitness-cost-incurring reactive oxygen species (ROS) resulting from overproduction of *CYP6ER1* and/or *CYP6AY1*.

## Results

### Selection of high- and low-resistant individuals of *N*. *lugens* for genome resequencing

Bioassay of the field population of *N*. *lugens* collected from Guangxi, China (called GX-P) in 2011 showed that it had a 50% lethal concentration (LC_50_) of 30.98 mg/l (S1 Table). Relative to the LC_50_ (0.08 mg/l or 0.28 mg/l) values of the 2 susceptible strains used by Wang et al. [37,46] and Wu et al. [39], GX-P was a field-evolved resistant population with a resistance level of 111- or 387-fold to imidacloprid (S1 Table). Such a resistance fold increase overlaps with the stable level of imidacloprid resistance (100- to 230-fold) in the absence of this insecticide [46], suggesting that the high-resistance individuals of GX-P most likely carried the unidentified fitness-improving allele(s) of the modifier gene(s).

To discriminate and obtain the high- (called GX-P-HR) and low-resistant (called GX-P-LR) individuals from this strain, we subjected the third instar nymphs of GX-P to the estimated LC_10_ (5 mg/l, the discriminating dose for GX-P-LR) or LC_90_ (300 mg/l, the discriminating dose for GX-P-HR) dose of imidacloprid (S2 Table). As expected, only 379 (12.03%) of the 3,150 tested nymphs survived the 300 mg/l imidacloprid treatment, and they were considered the GX-P-HR individuals. We randomly selected 2 replicates of 30 each from the 379 survivors for subsequent genomic DNA extraction and high throughput sequencing. By comparison, 5 mg/l imidacloprid killed 126 (7.64%) and 75 (7.14%) of the first (1,650) and second (1,050) batches of the tested nymphs. The dead nymphs of both batches were considered the GX-P-LR individuals, but the 2 replicates of 30 each used for subsequent genome resequencing were randomly selected from the nymphs that died on day 2 and 3 after exposure to imidacloprid (S2 Table).

### Mapping of the candidate adaptive loci for imidacloprid resistance

**Fig 1:**
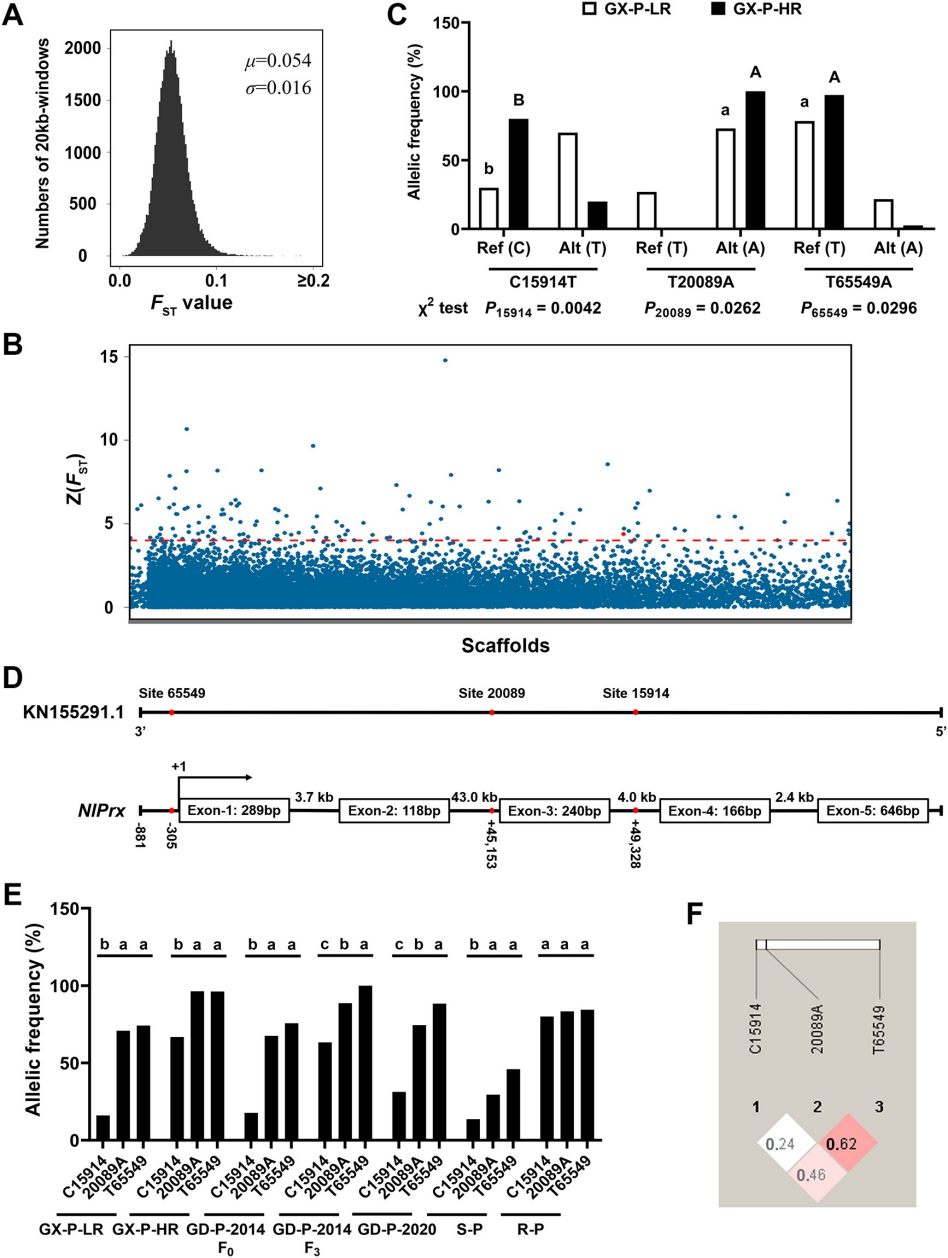
Identification of the putative modifier gene and its fitness-improving allele. (A) Distribution of the pairwise fixation index *F*_ST_ of 20-kb sliding windows between GX-P-HR and GX-P-LR. (B) Manhattan plot of the genome-wide *Z*-transformation of *F*_ST_, *Z*(*F*_ST_), between GX-P-HR and GX-P-LR. Windows that passed the threshold of *Z*(*F*_ST_) > 4 (the dashed horizontal line) were extracted as the candidate selective sweep regions. The sweep window with *NlPrx* is denoted by a red dot. (C) Estimated frequencies of the reference (Ref) and alternative (Alt) alleles of the 3 SNP sites (C15914T, T20089A, and T65549A) at the *NlPrx* locus with the sequence reads data from GX-P-LR and GX-P-HR. The allelic frequencies sharing the same letter within each subpopulation (capital letters for GX-P-HR and lowercase letters for GX-P-LR) are not significantly different at *P* < 0.05 (*χ*^2^ test). (D) Schematic alignment between *NlPrx* genomic sequence and scaffold KN155291.1. The exact locations of the 3 SNP sites are indicated by red dots in the *NlPrx* genomic sequence and scaffold KN155291.1, which are not drawn to scale. (E) Calculated frequencies of the C15914, 20089A, and T65549 alleles with genotyping data from GX-P-HR, GX-P-LR, S-P, R-P, GD-P-2014 F_0_, GD-P-2014 F_3_, and GD-P-2020. The allelic frequencies sharing the same letter within each subpopulation are not significantly different at *P* < 0.05 (*χ*^2^ test). (F) Linkage disequilibrium relationship among the C15914, 20089A, and T65549 alleles. The numbers in boxes show the D′ values between pairs of SNP alleles. Numerical values are provided in S1 Data (doi: 10.6084/m9.figshare.14177009).

Under field selection of imidacloprid, the alleles that confer adaptive advantages such as imidacloprid resistance and fitness improvement, along with their linked neighboring regions, are likely to have a significantly higher frequency in the GX-P-HR individuals than in the GX-P-LR individuals. To map out these genomic regions, we sequenced the genomes of the 2 pooled GX-P-HR and 2 pooled GX-P-LR DNA samples and aligned the obtained sequencing reads to the reference genome of *N*. *lugens* [47]. The resultant uniquely aligned sequencing reads covered approximately 94% of the reference genome of *N*. *lugens*, and yielded a coverage depth of approximately 31× for each of the 2 subpopulations (GX-P-HR and GX-P-LR) (S3 Table). This genome-wide alignment detected a total of 11,744,584 qualified SNPs (single nucleotide polymorphisms). The allele frequencies of all identified SNPs in the 2 replicates for GX-P-HR and GX-P-LR were highly correlated with each other (*R* ≥ 0.87; S1 Fig), validating the reliability of the resequencing data. The pairwise fixation index (*F*_ST_) values for each 20-kb sliding window between the GX-P-HR and GX-P-LR genomes, estimated using the above qualified SNPs, ranged from 0.000 to 0.294, with a mean value of 0.054 (Fig 1A). We *Z*-transformed the *F*_ST_ distribution and found 139 candidate selective sweep regions by applying a cutoff threshold of *Z*(*F*_ST_) > 4 (Fig 1B; S4 Table). A total of 69 genes were identified from these candidate selective sweep regions (S4 Table).

To select an adaptive locus for examining its contribution to reducing the fitness cost associated with imidacloprid resistance, we calculated the standardized difference (*D*) in Tajima’s *D* statistics [48] of the 139 candidate regions between GX-P-HR and GX-P-LR. Only 20 of the 69 candidate genes (67 of the 139 candidate regions) had a negative *D* value that signifies selective sweep genes (see column H in S4 Table). The top 6 most likely sweep genes were *dynamin* (*D* = −5.729), *transient receptor potential-gamma* (*D* = −3.986), *plexin* (*D* = −2.882), *glucose dehydrogenase* (*D* = −2.202), *anxa6 protein* (*D* = −1.779), and *peroxiredoxin* (*D* = −1.674) (S4 Table). The top 5 likely sweep genes have little chance to reduce fitness costs, because they are involved in clathrin-dependent endocytosis [49], sensation of visual cues [50], axonal pathfinding and epithelial wound healing [51], glucose oxidation [52], and endocytosis [53], respectively. By contrast, the sixth likely sweep gene, *N*. *lugens peroxiredoxin* (*NlPrx*, XLOC_032133), most likely acts as a fitness modifier. This is because Gene Ontology (GO) functional category analysis indicated that peroxiredoxin has molecular functions of peroxidase activity and antioxidant activity, which enable it to participate in biological processes related to cellular oxidant detoxification, response to toxic substances, detoxification, and cellular detoxification (S5 Table). The fact that insecticides including imidacloprid often enhance oxidative stress [54–56], which in turn reduces the fitness of insects [57,58], further supports our selection to examine *NlPrx* as a fitness-improving locus.

### Identification of the fitness-enhancing allele at the candidate modifier *NlPrx* locus

Mapping of GX-P-HR and GX-P-LR sequencing reads to the reference genome of *N*. *lugens* showed 3 significant SNPs out of 448 SNPs found at the candidate modifier *NlPrx* locus (*χ*^2^ test, *P* < 0.05; Fig 1C and S2 Fig). These included C15914T, T20089A, and T65549A (the nucleotides present in the reference genome and in the GX-P-HR and/or GX-P-LR individuals are placed before and after the position numbers, respectively). Hereafter, we refer to the reference (C15914, T20089, and T65549) and GX-P nucleotides (15914T, 20089A and 65549A) of the 3 SNP sites as the reference (ref) and alternative (alt) alleles, respectively. Counting the sequence reads aligned to the 3 SNP sites showed that the ref and alt alleles of the 3 SNP sites were not randomly distributed between the GX-P-HR and GX-P-LR individuals (Fig 1C). Instead, GX-P-HR had significantly higher frequencies of the ref (T65549 and C15914) or alt (20089A) alleles of the 3 SNP sites than did GX-P-LR, indicating that T65549, 20089A, and C15914 alleles were all genetically linked to imidacloprid resistance (Fig 1C). The nonrandom distribution of the 2 alleles at each of the 3 significant SNP sites of the *NlPrx* locus between GX-P-HR and GX-P-LR also suggested that *NlPrx* may play a role, either as a minor resistance gene or as a compensatory modifier gene, in resistance of *N*. *lugens* to imidacloprid.

Then, we obtained the full-length cDNA sequence of *NlPrx* (GenBank accession no. KR816210; S3 Fig) and aligned it to the genomic sequence of the *NlPrx* locus in scaffold KN155291.1. The alignment revealed that T65549, 20089A, and C15914 alleles are located in the regulatory promoter region (305 nucleotides upstream of the transcription site of *NlPrx*), intron 2 (45 kb downstream of T65549), and intron 3 (50 kb downstream of T65549), respectively (Fig 1D). According to the generalization that regulatory promoter sequences are more likely subject to natural selection than non-first introns [59,60], T65549 should be the adaptive sweep allele, whereas 20089A and C15914 should be the nearby neutral hitchhiker alleles. Consistent with this inference, in both the GX-P-HR and GX-P-LR subpopulations, the frequencies of the T65549 allele calculated by counting the reads mapped to the *NlPrx* locus were not different from those of the closer 20089A allele, but were significantly higher than the frequencies of the more distant C15914 allele (Fig 1C). Furthermore, genotyping of GX-P-HR, GX-P-LR, and other tested populations/subpopulations (S-P, R-P, GD-P-2014 F_0_, GD-P-2014 F_3_, and GD-P-2020) showed that the T65549 allele had the highest frequency, followed by the 20089A allele and the C15914 allele, in each of the 7 populations/subpopulations (Fig 1E). Linkage disequilibrium analysis of the genotyping data confirmed that the 3 alleles were in linkage disequilibrium, with a D′ value of 0.62, 0.46, and 0.24 for the T65549–20089A, T65549–C15914, and 20089A–C15914 allele pairs, respectively (Fig 1F).

### Genetic linkage of the *NlPrx* T65549 allele to imidacloprid resistance

**Fig 2:**
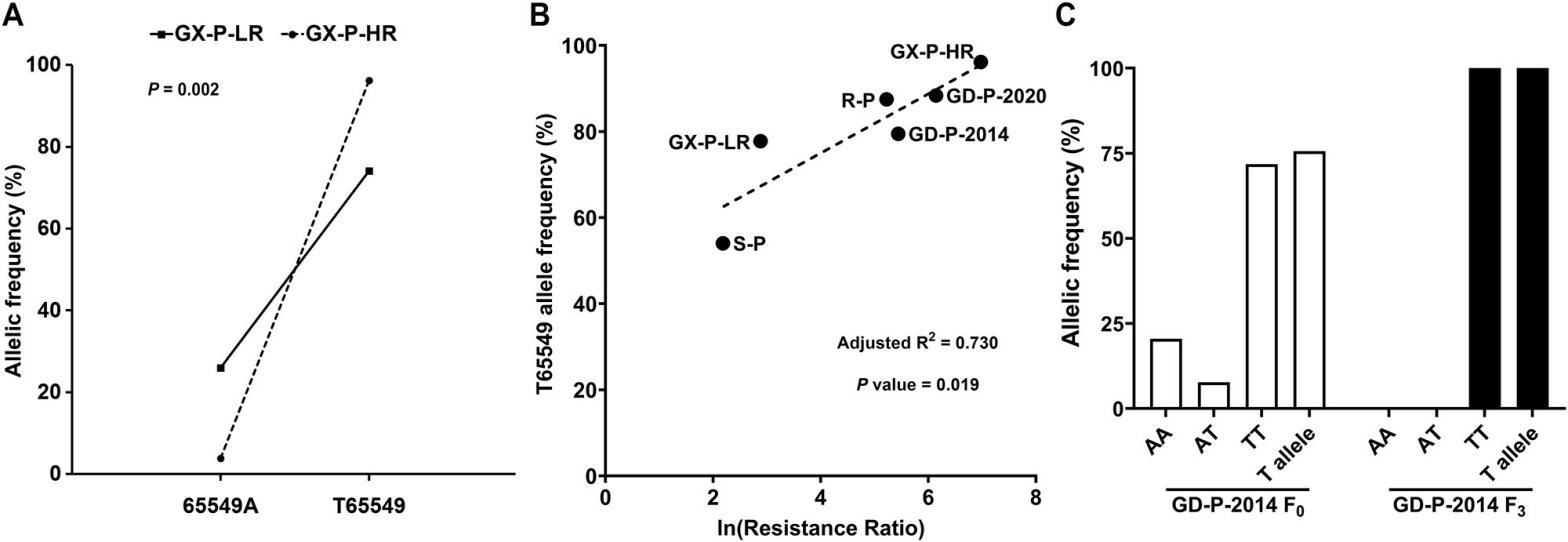
Correlation among *NlPrx* T65549 allele frequency, resistance level, and imidacloprid selection. (A) Significant correlation (*P* = 0.002) between the *NlPrx* T65549 allele and imidacloprid resistance in GX-P-LR and GX-P-HR. (B) Correlation between the *NlPrx* T65549 allele frequencies and the Ln-transformation of resistance ratios of 6 *N*. *lugens* populations/subpopulations. (C) Changes in the frequencies of the genotypes of the *NlPrx* 65549 SNP site and of the T65549 allele of GD-P-2014 before (F_0_) and after laboratory selection for 3 generations (F_3_) with 60 mg/l imidacloprid. Numerical values are provided in S1 Data (doi: 10.6084/m9.figshare.14177009).

Genotyping 27 GX-P-LR insects revealed 19 TT, 2 AT, and 6 AA individuals at the *NlPrx* 65549 SNP site. By contrast, genotyping of 26 GX-P-HR insects found 25 TT, 0 AT, and only 1 AA individual. Accordingly, the odds ratio of the likelihood of surviving exposure to imidacloprid for the TT genotype was significantly greater (*P* = 0.002) than that for the AA genotype (Fig 2A), suggesting a positive correlation between *NlPrx* T65549 allele frequency and imidacloprid resistance of *N*. *lugens*. We then bioassayed and genotyped 2 more field populations (GD-P-2014 and GD-P-2020, collected from Guangdong Province in 2014 and 2020, respectively) and 2 laboratory populations (the highly resistant R-P and the low-resistant S-P, provided by Zhejiang Academy of Agricultural Sciences) (S1 Table). Relative to the LC_50_ value of the susceptible strain used by Wu et al. [39], the 4 populations had resistance fold increases of 8.88 (S-P), 185.76 (R-P), 230.88 (GD-P-2014, F_0_ generation), and 466.07 (GD-P-2020) (S1 Table). Correlation analysis revealed that the resistance fold increases of the 4 populations as well as those of GX-P-LR and GX-P-HR were positively correlated with their T65549 allele frequencies (adjusted correlation coefficient *R*^2^ = 0.730, *P* = 0.019, Ln-transformation of resistance fold increase) (Fig 2B).

We also selected the GD-P-2014 population with 60 mg/l imidacloprid for 3 generations and genotyped the individuals randomly picked from the GD-P-2014 population before (F_0_ generation) and after (F_3_ generation) imidacloprid selection. Before selection, the GD-P-2014 population was composed of 71.8% homozygous TT (T65549), 20.5% homozygous AA (65549A), and 7.7% heterozygous TA (T65549/65549A) individuals. After selection, both homozygous AA and heterozygous TA individuals were eliminated, leaving homozygous TT individuals only (Fig 2C). Accordingly, imidacloprid selection drove the T65549 allele from a frequency of 75.6% in the F_0_ generation to fixation (100%) in the F_3_ generation (*P* = 8.125 × 10^−6^, *χ*^2^ test).

### Phenotypic effects of the T65549 allele

**Fig 3:**
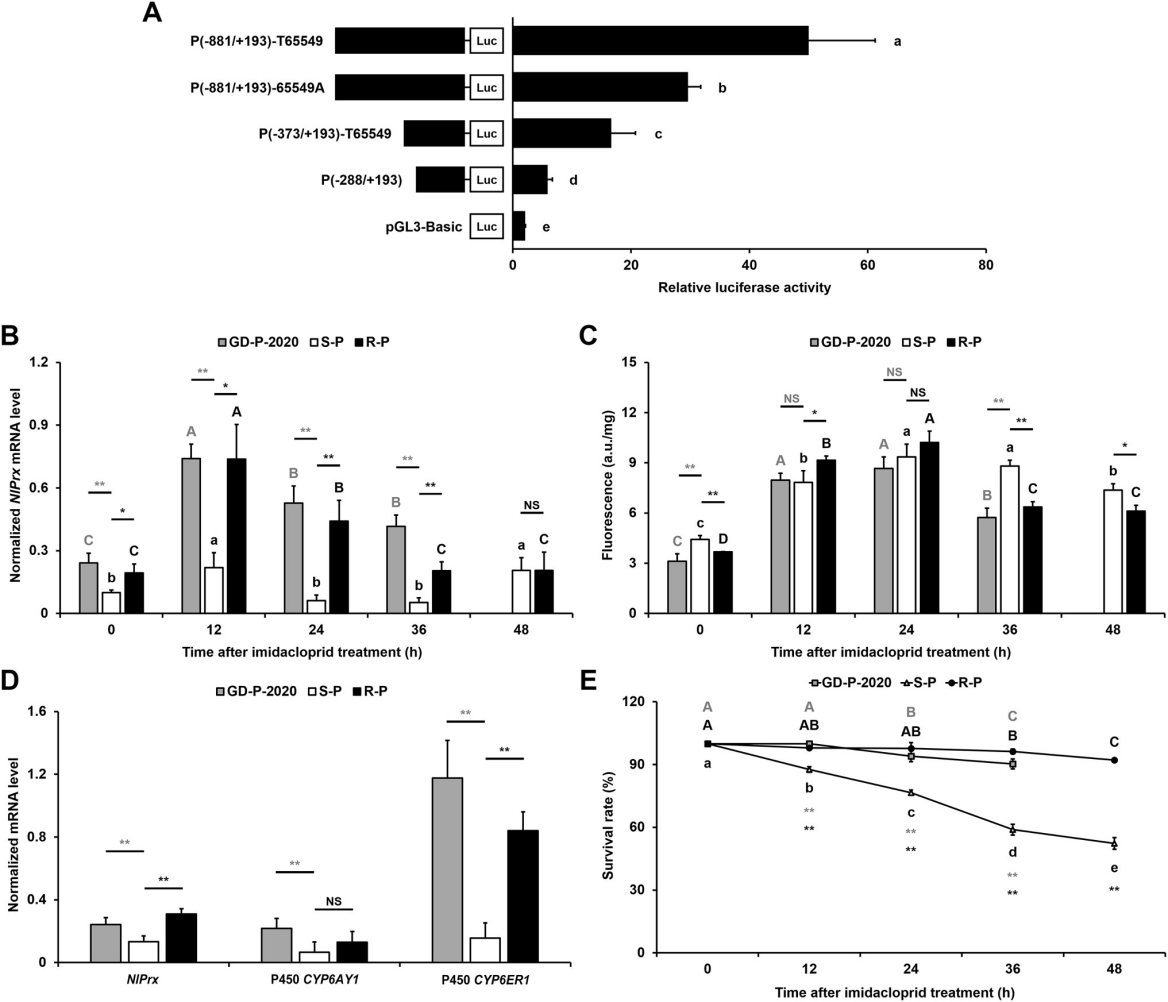
Impacts of *NlPrx* T65549 allele on *NlPrx* expression, ROS level, and survivorship in the absence and presence of imidacloprid. (A) The contribution of the T65549 allele to the promoter activity of *NlPrx*. The numbers in parentheses of the *NlPrx* promoter constructs specify the beginning and end positions of the promoter fragments. (B) Time course of *NlPrx* expression in GD-P-2020, S-P, and R-P in response to 10 mg/l imidacloprid. (C) Time course of the ROS burst elicited by 10 mg/l imidacloprid in GD-P-2020, S-P, and R-P. (D) Constitutive expression levels of *NlPrx*, *CYP6ER1*, and *CYP6AY1* in GD-P-2020, S-P, and R-P. Data represent the mean value ± SD of 3 biological replicates (*n* ≥ 3). (E) Time course of the survival rate with 10 mg/l imidacloprid in GD-P-2020, S-P, and R-P. Values sharing the same letter (lowercase letters for S-P and capital letters for GD-P-2020 and R-P) are not significantly different at *P* < 0.05 (1-way ANOVA and Duncan’s multiple range test). Extremely significant (*P* < 0.01) and significant (*P* < 0.05) differences between GD-P-2020/R-P and S-P (B–E) or the 2 promoter constructs (A) are depicted by double and single asterisks (Student *t* test), respectively. NS, no significant difference; ROS, reactive oxygen species. Numerical values are provided in S1 Data (doi: 10.6084/m9.figshare.14177009).

To test if enhancing the expression of *NlPrx* is the proximal phenotypic effect of the T65549 allele, we made 2 full-length pGL3-*NlPrx* promoter constructs (P[−881/+193]-T65549 and P[−881/+193]-65549A) and 2 5′ progressive deletion constructs (P[−373/+193]-T65549 and P[−288/+193]) and tested their constitutive promoter activities in *Trichoplusia ni* (Hi5) cells. The full-length T65549 allele promoter construct P(−881/+193)-T65549 had the highest promoter activity, followed by the full-length 65549A allele promoter construct P(−881/+193)-65549A (its promoter activity was 59.17% of the T65549 allele construct, *P* < 0.05), the 65549-site-containing deletion construct P(−373/+193)-T65549, and the 65549-site-free deletion construct P(−288/+193) (its promoter activity was 35.34% of P[−373/+193]-T65549, *P* < 0.05) (Fig 3A). These results suggested that the T allele at 65549 was responsible for constitutive overexpression of *NlPrx*.

We then compared the time courses of *NlPrx* expression, ROS production (a possible source of the fitness cost associated with imidacloprid resistance), and survivorship between R-P, a high-resistant population with a T65549 allele frequency of 84.38%, and S-P, a low-resistant population with a T65549 allele frequency of 45.95% (S1 Table; Fig 2B), after exposure to 10 mg/l imidacloprid. In the absence of imidacloprid (0 time point), R-P had a significantly higher level of *NlPrx* expression but a significantly lower level of ROS than did S-P (Fig 3B and 3C), consistent with the ROS-scavenging function of NlPrx [61]. Exposure to imidacloprid rapidly induced the expression of *NlPrx* to its highest level at 12 h in both populations, but the magnitude of the induction was significantly higher in R-P than in S-P (Fig 3B). The induction of *NlPrx* expression lasted only for 12 h and reappeared at 48 h in S-P, but persisted for 24 h and faded at 36 and 48 h in R-P. Except for the 48-h time point, when the expression level of *NlPrx* was similar in the 2 populations, the expression level of *NlPrx* in R-P was consistently higher than that of *NlPrx* in S-P (Fig 3B).

Meanwhile, exposure to imidacloprid elicited a >48-h ROS burst consisting of an ascending phase and a descending phase, which were located on the left and right sides of the 24-h peak, respectively, in both S-P and R-P (Fig 3C). At the 24-h peak, the turning point from the early ascending phase to the subsequent descending phase, the ROS levels in R-P (ROS_R-P_) and S-P (ROS_S-P_) were equal. By contrast, the ROS level was significantly higher during the ascending phase (at 12 h) but significantly lower during the descending phase (at 36 and 48 h) in R-P than in S-P (Fig 3C). The time course of ROS_R-P_ minus ROS_S-P_ above implies that 2 opposing mechanisms—one that generates ROS and another that scavenges ROS—worked in concert to cope with the imidacloprid challenge in both populations, and both mechanisms were stronger in R-P than in S-P. The correlation of the gradual decline of ROS_R-P_ minus ROS_S-P_ (Fig 3C) with the greater/longer induction of *NlPrx* and its consistently higher expression in R-P (Fig 3B) suggests that NlPrx is responsible for ROS removing, which is one of the distal phenotypic effects of the T65549 allele.

The contribution of overexpression of *CYP6ER1* and/or *CYP6AY1* to imidacloprid resistance in *N*. *lugens* [40–44] and the ability of P450s to generate ROS while metabolizing xenobiotics [62–64] led us to assume that the greater induction of ROS by imidacloprid in R-P than in S-P during the early ascending phase (Fig 3C) was due to the higher expression of *CYP6ER1* and/or *CYP6AY1* in R-P. Quantitative analyses of the 2 P450 genes and *NlPrx* revealed significantly higher basal expressions of *CYP6ER1* and *NlPrx* but not *CYP6AY1* in R-P than S-P (Fig 3D).

At the organismal level, exposure to 10 mg/l imidacloprid killed significantly more S-P individuals than R-P individuals at every time point post-exposure (Fig 3E). No significant reduction in the survivorship of R-P was observed until 36 h post-exposure, and about 92% of R-P individuals remained alive at the end of this experiment. By contrast, the survivorship of S-P rapidly and dramatically declined from 12 h post-exposure onwards, with only 48% of individuals alive at the end of this experiment (Fig 3E).

Similarly, we also compared the time courses of *NlPrx* expression, ROS production, and survivorship as well as the constitutive expression of *NlPrx*, *CYP6AER1*, and *CYP6AY1* in GD-P-2020, a fresh field resistant subpopulation with a T65549 allele frequency of 88.37% (S1 Table), to those in S-P (Fig 3B–3E), after exposure to 10 mg/l imidacloprid. Pairwise comparison showed that GD-P-2020 and S-P exhibited a similar difference profile as R-P and S-P (Fig 3B–3E).

### Contribution of the *NlPrx* T65549 allele to reducing the fitness cost of imidacloprid resistance

**Fig 4:**
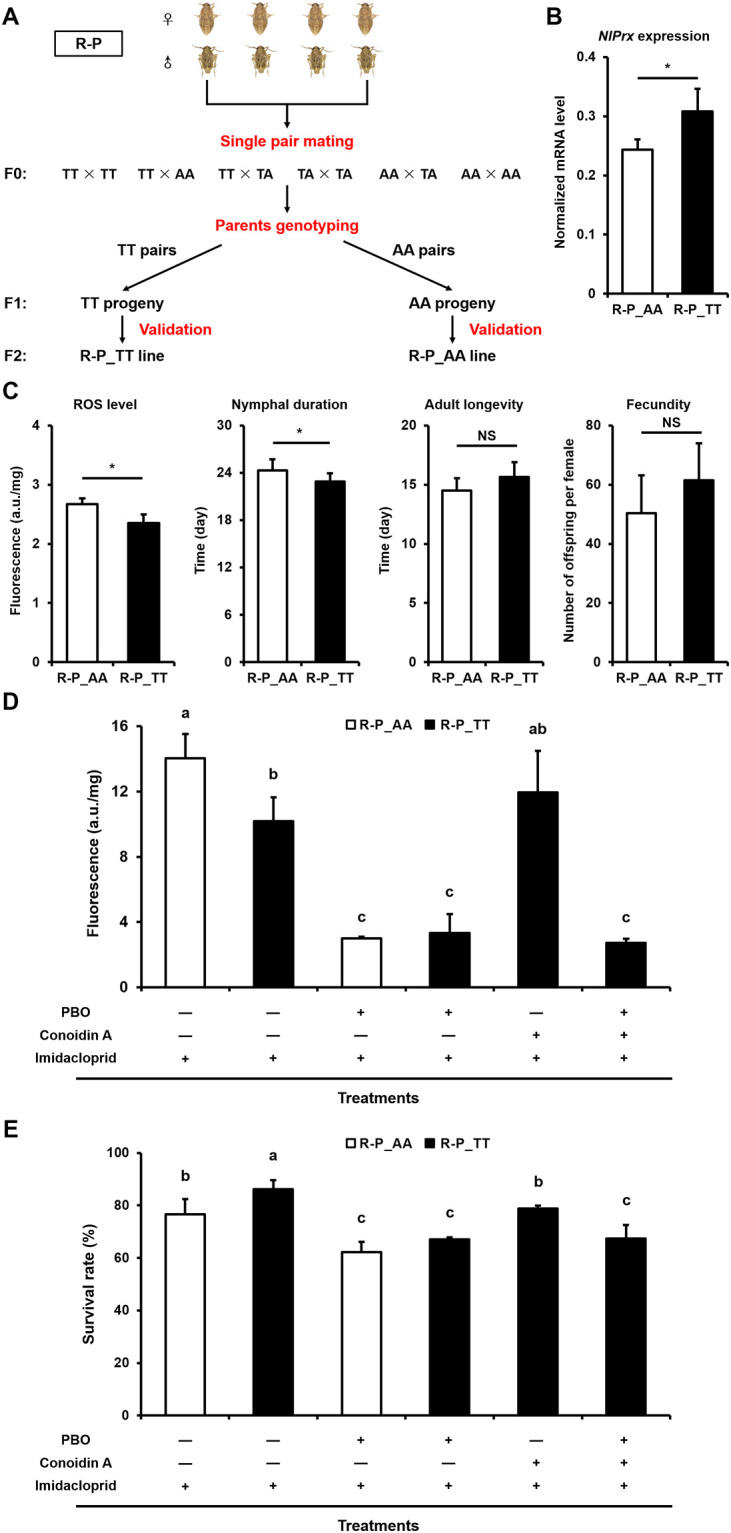
Comparison of the life history traits, ROS levels, and survivorship of the R-P_TT and R-P_AA isogenic lines under different treatment regimes. (A) Schematic diagram for establishment of R-P_TT (with homozygous *NlPrx* T65549 alleles) and R-P_AA (with homozygous *NlPrx* 65549A alleles) isogenic lines from the highly resistant R-P population. (B) The *NlPrx* mRNA level of R-P_TT was compared to that of R-P_AA. (C) Comparison of the life history traits and ROS levels of the R-P_TT and R-P_AA lines under normal rearing conditions. (D) Comparison of the ROS levels of the R-P_TT and R-P_AA lines treated with 10 mg/l imidacloprid alone, 10 mg/l imidacloprid + 5 mg/l PBO, 10 mg/l imidacloprid + 0.1 mmol *Conoidin A*, or 10 mg/l imidacloprid + 5 mg/l PBO + 0.1 mmol *Conoidin A*. For all the mixture treatments, 1-day-old female adults of both lines were pre-exposed to PBO and/or *Conoidin A* for 5 h and then exposed to the corresponding mixture for 19 h. (E) Comparison of the corrected survival rates of the R-P_TT and R-P_AA lines treated with 10 mg/l imidacloprid alone, 10 mg/l imidacloprid + 5 mg/l PBO, 10 mg/l imidacloprid + 0.1 mmol *Conoidin A*, or 10 mg/l imidacloprid + 5 mg/l PBO + 0.1 mmol *Conoidin A*. Data represent the mean value ± SD of 3 biological replicates (*n* ≥ 3). The values denoted with different lowercase letters are significantly different at *P* < 0.05 (1-way ANOVA and Duncan’s multiple range test). Significant differences between R-P_TT and R-P_AA are depicted by an asterisk (Student *t* test). NS, no significant difference; PBO, piperonyl butoxide; ROS, reactive oxygen species. Numerical values are provided in S1 Data (doi: 10.6084/m9.figshare.14177009).

To determine whether the T65549 allele acts as a fitness cost modifier allele for resistance of *N*. *lugens* to imidacloprid, we generated 2 isogenic lines—one with homozygous T65549 alleles (R-P_TT line) and the other with homozygous 65549A alleles (R-P_AA line)—from the *CYP6ER1*-mediated imidacloprid-resistant strain R-P (Fig 4A). As expected, the basic mRNA level of *NlPrx* was significantly higher in the R-P_TT line than in the R-P_AA line (1.27-fold) (Fig 4B). In the absence of imidacloprid, the ROS level was significantly lower in the R-P_TT line than in the R-P_AA line (0.88-fold) (Fig 4C). In addition, the R-P-TT line displayed a significantly shorter duration of nymph development, a numerically longer adult life span, and a numerically greater fecundity, relative to the R-P-AA line (Fig 4C).

When treated with 10 mg/l imidacloprid for 24 h, both lines experienced a dramatic ROS burst (Fig 4D), relative to their ROS levels in the absence of imidacloprid (Fig 4C). While the ROS level was still significantly lower in the R-P_TT line than in the R-P_AA line, the between-line difference expanded from 0.88-fold in the absence of imidacloprid to 0.72-fold in the presence of imidacloprid. When the 2 isogenic lines were simultaneously treated with the general P450 monooxygenase inhibitor piperonyl butoxide (PBO, 5 mg/l) and imidacloprid (10 mg/l), neither of the 2 isogenic lines underwent the imidacloprid-elicited ROS burst, and no significant difference in the ROS level was found between the 2 isogenic lines (Fig 4D). By contrast, when the R-P_TT line was simultaneously treated with the peroxiredoxin II inhibitor (NlPrx belongs to peroxiredoxin II) *Conoidin A* (0.1 mmol) [65] and imidacloprid (10 mg/l), the R-P_TT line exhibited a ROS burst that was marginally larger than that of the R-P_TT line treated with imidacloprid alone but marginally lower than that of the R-P-AA line treated with imidacloprid alone. Furthermore, the R-P_TT line treated with PBO + *Conoidin A +* imidacloprid had the lowest ROS level, which was not significantly different from those of the R-P_TT and R-P_AA lines treated with PBO + imidacloprid (Fig 4D).

Multiple comparison showed that the R-P_TT line exhibited a significantly higher survival rate than the R-P_AA line when they were exposed to imidacloprid alone (Fig 4E). Exposure to PBO + imidacloprid resulted in a significantly lower survival rate in both isogenic lines, relative to imidacloprid alone. No significant differences in the survival rate were found between the 2 lines treated with PBO + imidacloprid. Among the 4 treatments in the R-P_TT line, imidacloprid alone yielded the highest survival rate, followed by *Conoidin A +* imidacloprid, and then PBO + imidacloprid and PBO + *Conoidin A +* imidacloprid. There was no significant difference in the survival rate between PBO + imidacloprid and PBO + *Conoidin A +* imidacloprid (Fig 4E).

### Functional confirmation of NlPrx in reducing ROS cost

**Fig 5:**
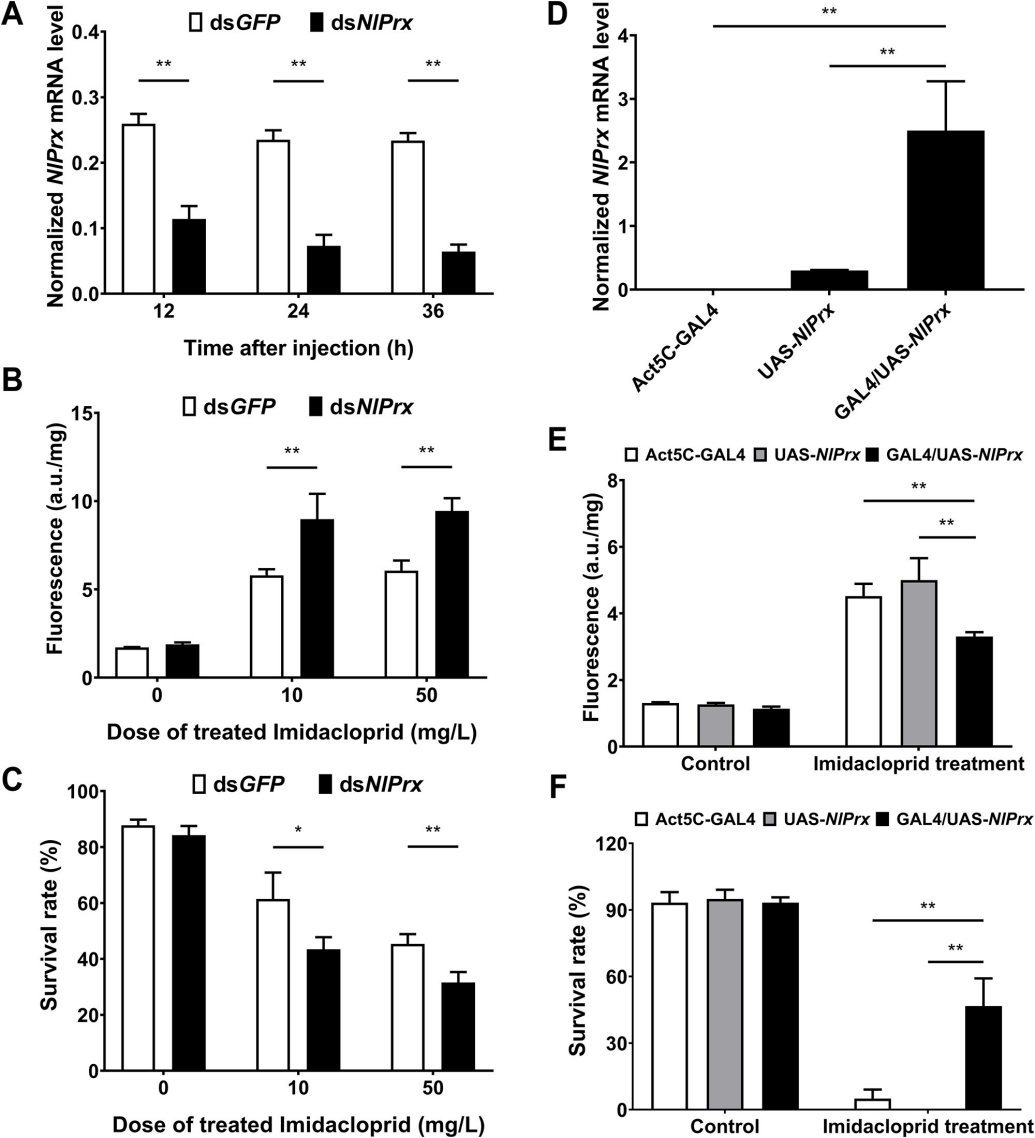
Impacts of RNAi-mediated knockdown and heterologous expression of *NlPrx* on the ROS levels and survival rates of R-P adults of *N*. *lugens* or the GAL4/UAS-*NlPrx* progeny line of *D*. *melanogaster*. (A) Time course of the expression level of *NlPrx* in R-P adults injected with ds*GFP* (control) or ds*NlPrx*. (B) Impacts of 10 or 50 mg/l imidacloprid on the ROS levels of R-P adults pre-injected with ds*GFP* (control) or ds*NlPrx*. (C) Impacts of 10 or 50 mg/l imidacloprid on the survival rates of R-P adults pre-injected with ds*GFP* (control) or ds*NlPrx*. (D) qRT-PCR detection of *NlPrx* transcripts in the GAL4/UAS-*NlPrx* progeny line, UAS-*NlPrx* transgenic line, and Act5C-GAL4 driver line of *D*. *melanogaster*. (E) Impacts of 30 mg/l imidacloprid on the ROS levels of the GAL4/UAS-*NlPrx* progeny line, UAS-*NlPrx* transgenic line, and Act5C-GAL4 driver line of *D*. *melanogaster*. (F) Impacts of 30 mg/l imidacloprid on the survival rates of the GAL4/UAS-*NlPrx* progeny line, UAS-*NlPrx* transgenic line, and Act5C-GAL4 driver line of *D*. *melanogaster*. The R-P adults injected with ds*NlPrx* or ds*GFP* were exposed to different doses (0, 10, or 50 mg/l) of imidacloprid 24 h after injection of dsRNA. The ROS levels and survival rates of these adults and the 3 lines of *D*. *melanogaster* were measured 24 and 48 h after exposure to imidacloprid, respectively. All data shown are mean ± SD of 3 biological replicates (*n* ≥ 3). Extremely significant (*P* < 0.01) and significant (*P* < 0.05) differences are depicted by double and single asterisks (Student *t* test), respectively. NS, no significant difference; qRT-PCR, quantitative reverse transcription PCR; ROS, reactive oxygen species; RNAi, RNA interference. Numerical values are provided in S1 Data (doi: 10.6084/m9.figshare.14177009).

We performed a loss-of-function analysis by RNA interference (RNAi) of the endogenous *NlPrx* in the R-P strain of *N*. *lugens*. Quantitative reverse transcription PCR (qRT-PCR) analysis showed that injection of the double-stranded (ds) RNA of *NlPrx* (ds*NlPrx*) effectively and significantly reduced the expression of the endogenous *NlPrx* in R-P adults, with a silencing efficiency of about 60% (12 h post-injection) and 70% (24 and 36 h post-injection), compared with injection of ds*GFP* (Fig 5A). We further exposed the dsRNA-injected R-P individuals to 10 and 50 mg/l of imidacloprid at 24 h post-injection, measured their ROS levels at 24 h after imidacloprid exposure, and recorded their survival at 48 h after imidacloprid treatment. As expected, insects injected with ds*NlPrx* had a significantly higher level of ROS than those injected with ds*GFP* at both doses (1.55-fold at 10 mg/l [*P* = 0.020] and 1.56-fold at 50 mg/l [*P* = 0.003]) (Fig 5B). Additionally, there was a significant decline in the survival rate of the insects injected with ds*NlPrx* (45.35% at 10 mg/l and 31.57% at 50 mg/l), compared with those injected with ds*GFP* (61.43% at 10 mg/l [*P* = 0.014] and 43.44% at 50 mg/l [*P* = 0.002]) (Fig 5C).

Meanwhile, a gain-of-function analysis by heterologous expression of *NlPrx* was performed in *D*. *melanogaster*. Reverse transcription PCR (RT-PCR) (S4 Fig) and qRT-PCR (Fig 5D) confirmed the absence of *NlPrx* transcripts in the Act5C-GAL4 driver line, a very low level of leaked expression of *NlPrx* in the UAS-*NlPrx* reporter line, and the strongest expression of *NlPrx* in the GAL4/UAS-*NlPrx* line, the F_1_ progeny of Act5C-GAL4 line × UAS-*NlPrx* line. In contrast to the expression level of *NlPrx* among the 3 *D*. *melanogaster* lines, the GAL4/UAS-*NlPrx* progeny line had the lowest ROS level, followed by the UAS-*NlPrx* reporter line and the Act5C-GAL4 driver line, both in the absence (control) and presence of imidacloprid (Fig 5E). By contrast, the survival rate from exposure to 30 mg/l imidacloprid was significantly higher (46.67%) in the GAL4/UAS-*NlPrx* progeny line than in the UAS-*NlPrx* reporter line (0%) and the Act5C-GAL4 driver line (5.0%) (Fig 5F).

## Discussion

Whole-genome sequencing and sweep mapping of individuals with high (GX-P-HR) and low (GX-P-LR) resistance to imidacloprid from the field-evolved resistant population GX-P yielded 139 candidate selective sweep regions that hosted 69 genes (S4 Table). Further analyses of Tajima’s *D* statistics (column H in S4 Table) and of GO functional categories (S5 Table) of the 69 candidate genes between GX-P-HR and GX-P-LR identified *NlPrx* as the most likely fitness modifier gene candidate of imidacloprid resistance in *N*. *lugens*. This agrees with the function of peroxiredoxins to scavenge ROS, which can be elicited by exposure to many insecticides and plant defense allelochemicals [61,54–56,66–68] and can reduce the fitness of insects [57,58]. This is also consistent with the findings that field-evolved imidacloprid resistance in *N*. *lugens* is mainly conferred by overexpression of the P450 genes *CYP6ER1* and *CYP6AY1* [40–44,69] and that P450s are known to be capable of generating ROS while metabolizing xenobiotics [62–64,69–73].

Our initial finding of nonrandom distribution of the 2 alleles at each of the 3 significant SNP sites of the *NlPrx* locus between GX-P-HR and GX-P-LR (Fig 1C) suggests that *NlPrx* may play a role in resistance of *N*. *lugens* to imidacloprid. Among the 3 alleles (T65549, 20089A, and C15914) that have a significantly higher frequency in GX-P-HR than GX-P-LR, the T65549 allele is the adaptive sweep allele selected by field application of imidacloprid, whereas the 20089A and C15914 alleles are the nearby neutral hitchhikers of the T65549 allele. This is evidenced by their locations within *NlPrx* (promoter versus intron 2 or 3) and the conception that promoters are more likely subject to natural selection than non-first introns [59,60]. Additional evidence includes the higher frequency of the T65549 allele than the other 2 alleles in each of the 7 genotyped populations/subpopulations (GX-P-HR, GX-P-LR, S-P, R-P, GD-P-2020, GD-P-2014 F_0_, and GD-P-2014 F_3_) with a different level of imidacloprid resistance (S1 Table; Fig 1E) and the linkage disequilibrium relationship among the 3 alleles (Fig 1F).

The positive correlation between T65549 allele frequencies and resistance fold increases of the tested populations/subpopulations as well as the imidacloprid-driven fixation of the T65549 allele in the GD-P-2014 F_3_ subpopulation (Fig 2) indicate that the T65549 allele is phenotypically beneficial to *N*. *lugens* individuals with imidacloprid resistance. The direct and proximal phenotypic effect of the T65549 allele is to enhance the expression of *NlPrx* both in the absence and presence of imidacloprid (Fig 3). The significantly lower ROS level but higher *NlPrx* expression at 0 and 36 h after imidacloprid exposure in the high-resistant R-P population than in the low-resistant S-P population suggest that scavenging ROS is the direct phenotypic effect of the upregulation of *NlPrx* by the T65549 allele. The phenotypic effect of clearing ROS is not only consistent with the ROS-scavenging function of peroxiredoxins [61,66,67], but is also supported by the ROS burst elicited by imidacloprid in R-P and S-P. Additionally, the fact that the high-resistant R-P population, with overexpression of *CYP6ER1* and *NlPrx*, displayed an imidacloprid-elicited ROS burst consisting of a larger ascending phase but a smaller descending phase relative to the low-resistant S-P population (Fig 3C) suggests that CYP6ER1 is a pleiotropic P450 that generates ROS when detoxifying imidacloprid, whereas NlPrx functions to reduce the ROS associated with CYP6ER1-mediated imidacloprid resistance. Our subsequent experiments in the GD-P-2020 population, which was freshly collected from the field, further demonstrated the field relevance of this phenomenon (Fig 3).

The data obtained with the isogenic R-P_TT and R-P_AA lines derived from the *CYP6ER1*-mediated resistant strain R-P directly confirm that CYP6ER1 produces ROS when metabolizing imidacloprid and its other substrates, whereas *NlPrx* serves as a modifier gene to clear ROS (Fig 4). First, the lower ROS level exhibited by R-P_TT relative to R-P_AA in the absence and presence of imidacloprid indicates that the NlPrx upregulated by the T65549 allele indeed clears the ROS resulting from the metabolism of imidacloprid or other compounds present in the rice plants that we used to feed *N*. *lugens*. The concurrent reduction of ROS and survival rate to a similar level in the 2 isogenic lines with imidacloprid + P450 inhibitor PBO treatment not only confirms that the imidacloprid resistance in R-P is conferred by CYP6ER1 alone, not by CYP6ER1 plus NlPrx, but also demonstrates that the ROS burst is not generated by imidacloprid itself, but by CYP6ER1-mediated metabolism of imidacloprid. Additionally, the fact that the inhibition of NlPrx in R-P_TT by imidacloprid + peroxiredoxin II inhibitor *Conoidin A* led to an increased ROS level and lower survival rate relative to imidacloprid alone verifies that NlPrx overcomes the fitness cost through scavenging the ROS resulting from the metabolism of imidacloprid by CYP6ER1. RNAi silencing of the endogenous *NlPrx* in R-P as well as heterologous expression of *NlPrx* in *D*. *melanogaster* (Fig 5) further confirm that NlPrx functions to clear ROS and protect resistant insects against the inevitable production of ROS associated with P450-mediated metabolism of imidacloprid or other chemicals present in the host plants. These data also demonstrate that *NlPrx* contributes, as a modifier gene rather than a minor resistant gene, to the resistance of *N*. *lugens* to imidacloprid because PBO inhibition of CYP6ER1 abolished its roles of ROS removal and protection of R-P individuals (Fig 4).

Our experiments have validated that *NlPrx* acts as a modifier gene for ROS cost associated with imidacloprid resistance in *N*. *lugens*, but we cannot exclude that additional genes from the 69 candidates (S4 Table) may also serve as fitness modifiers, particularly the serine/threonine protein kinase *ulk2-like* (No. XLOC_031284), a mitogen-activated protein kinase (MAPK) gene. On the one hand, MAPK genes have been recently proved to indirectly confer Cry1Ac resistance by downregulating Cry1Ac receptors in the diamondback moth [33,74] and imidacloprid resistance by upregulating the P450 gene *CYP6CM1* in the whitefly [11]. One the other hand, MAPK genes have been speculated to mediate the balanced rise of both juvenile hormone (JH) and molting hormone (20E), leading to reduction in the fitness cost incurred by Cry1Ac resistance in the diamondback moth [33]. Additionally, the generation of ROS and activation of MAPK members are both observed in insects under xenobiotic stress [75,76], suggesting that the ROS-scavenging system and MAPK-dependent pleiotropic hormone signaling network may work together to minimize the fitness costs of insecticide resistance. Further research is needed to reveal if the 2 modifier systems coexist and how they are orchestrated to reduce fitness cost when both modifier systems are present.

**Fig 6:**
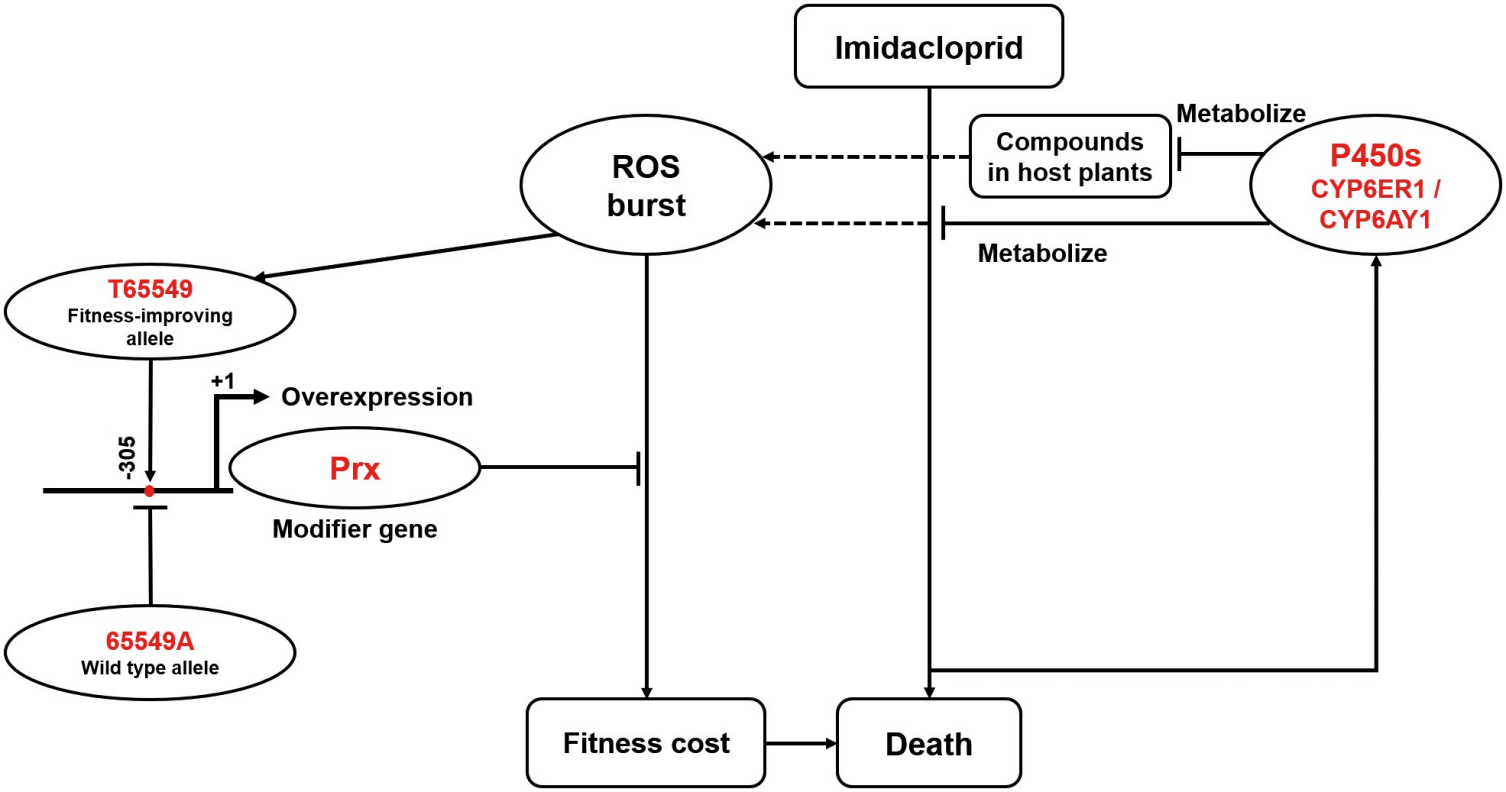
A working model for the sequential evolution of the overexpression alleles of P450s and *NlPrx* as well as their contributions to resistance of *N*. *lugens* to imidacloprid. →, stimulation; ⟞, inhibition; Prx, peroxiredoxin; ROS, reactive oxygen species.

Continued use of an insecticide following emergence of resistance is suggested to drive evolution of modifier genes/alleles in theory, but examples have rarely been reported [15,29]. Our discovery that the T65549 allele upregulated expression of NlPrx to clear ROS of any source and to reduce the fitness cost (Fig 6) represents to our knowledge the first functional characterization of such fitness-improving modifier genes/alleles. We showed that the fitness cost of imidacloprid resistance in *N*. *lugens* results from the inevitable production of ROS by the resistance-conferring P450 CYP6ER1 (and to a lesser extent, CYP6AY1) through metabolism of imidacloprid and/or host plant compounds (Fig 6). This provides insight into the scientific question of how a nontarget resistant gene/allele incurs a corresponding fitness cost. Nonetheless, this does not necessarily refute the hypothesis that overproduction of detoxification enzymes takes resources and energy away from growth, development, and/or reproduction [14]. The fact that P450s are the primary superfamily of detoxification enzymes associated with resistance to most insecticides [7,77] and that many insecticides can induce production of ROS [62,78,79] suggests that *Prx* or other ROS-scavenging genes may serve as potential common modifier genes for overcoming the fitness cost of insecticide resistance. Quantitative analyses of the expression of *Prx* and other ROS-scavenging genes in a wide range of insects with P450-mediated insecticide resistance are necessary to resolve this speculation.

## Methods

### Insects

A total of 5 populations of *N*. *lugens* were used in this study: GX-P, GD-P-2014, GD-P-2020, R-P, and S-P. The GX-P population used for genome resequencing and sweep mapping was established in 2011 by pooling more than 500 adults randomly sampled from fields in Guangxi, China. The heterogeneous GD-P-2014 population collected in the field of Guangdong Province, China, in 2014 was used to verify if the candidate modifier locus *NlPrx* is under positive selection by laboratory selection with imidacloprid (60 mg/l) and genotyping. The high-resistant R-P (LC_50_ 52.02 mg/l) and low-resistant S-P (LC_50_ 2.49 mg/l) populations provided by Zhejiang Academy of Agricultural Sciences, and a field population (GD-P-2020) collected from Shaoguan, Guangdong Province, China in August 2020, were used for several comparative experiments to link allele frequency with NlPrx expression, ROS level, resistance fold increases, and fitness in the presence and absence of imidacloprid. All these populations/subpopulations were reared on “Fenghuazhan” (a brown-planthopper-susceptible variety) rice plants in a controlled environment (25 ± 2 °C and a 14-h light/10-h dark photoperiod).

### Toxicity bioassays

We bioassayed the susceptibilities of GX-P, GD-P, R-P, and S-P to imidacloprid using the rice stem dipping method [41]. Briefly, imidacloprid dissolved in acetone was used as the stock solution to prepare at least 7 serially diluted solutions with double-distilled water. Rice stems rooted in culture cups were then dipped into the imidacloprid solutions or acetone water solution (control) for 30 s. After the rice stems were air dried, 20 third instar nymphs were put into each culture cup. Each concentration or control was performed in triplicate of 20 nymphs (*N* = 60). Nymph death was observed and recorded 96 h post-treatment. The lethal concentration–mortality probit regression line (LC-P line) and LC_50_ of each population were estimated using probit analysis in SAS (version 9.2, SAS Institute, Cary, NC, US). The resistance ratio of each population was calculated by dividing the LC_50_ of each population with the LC_50_ of the 2 susceptible strains used by Wang et al. [37,46] and Wu et al. [39].

According to the LC-P line and LC_50_ values of GX-P obtained above, we exposed the third instar nymphs of the second generation of GX-P to 300 mg/l (approximately a LC_90_ dose) and 5 mg/l imidacloprid (approximately a LC_10_ dose). The nymphs that survived LC_90_ treatment were considered highly resistant (GX-P-HR) individuals, whereas those that died from LC_10_ treatment were considered low-resistant (GX-P-LR) individuals.

### Genome resequencing of GX-P and sweep mapping analysis

Two replicates of 30 individuals randomly selected from GX-P-HR or GX-P-LR were used to extract genomic DNA samples as previously described [41]. The whole genome of the 2 DNA samples of GX-P-HR and GX-P-LR were sequenced on the Illumina HiSeq 2000 platform at BGI-Shenzhen (Shenzhen, China), producing 100-bp paired-end reads. The high-quality reads were aligned to the reference genome of *N*. *lugens* (GenBank Accession No. GCF_000757685.1) [47] using BWA 0.6.2-r126 [80], and SNP-based variants were called across samples using the Genome Analysis Toolkit (GATK) [81]. Subsequently, the genetic diversities were estimated by calculating pairwise *F*_ST_ values in a sliding window manner. Because the scaffold N90 of *N*. *lugens* is approximately 45 kb, we calculated the average *F*_ST_ within a nonoverlapping 20-kb window. Contigs less than 10 kb were excluded from our analysis. We then *Z*-transformed the *F*_ST_ values, and the outliers with a *Z*(*F*_ST_) score of >4 were considered as putative sweep regions selected by the application of imidacloprid. The Tajima’s *D* was calculated for each of GX-P-LR and GX-P-HR using a nonoverlapping 20-kb sliding window size by VCFtools [82]. Because we were aimed at finding regions of the genome that have been subject to recent positive selection in GX-P-HR, we further compared Tajima’s *D* between GX-P-HR and GX-P-LR using the standardized difference of *D*, describing the difference of Tajima’s *D* between 2 populations, as was described by Bigham et al. [48].

### Laboratory selection of GD-P with imidacloprid and *NlPrx* genotyping

The third instar nymphs of GD-P were selected for 3 generations with 60 mg/l (approximately a LC_50_ dose) imidacloprid in the laboratory. Nymphs randomly picked from the *F*_0_ (before selection) and *F*_3_ generations were individually genotyped to monitor the dynamics of the SNP alleles at the *NlPrx* locus by PCR sequencing. The primers for PCR amplification of the 3 SNP sites are listed in S6 Table. All PCR products were sent for Sanger sequencing by Invitrogen (Carlsbad, CA, US). In addition, we used the same method to genotype nymphs randomly picked from each of the 4 populations GX-P-HR, GX-P-LR, S-P, and R-P. After genotyping, we calculated the D′ value between pairs of SNP alleles using Haploview software version 4.2 to estimate the linkage disequilibrium patterns among the alleles of the 3 SNP sites within the *NlPrx* locus [83].

### Characterization of *NlPrx*

5′ and 3′ RACE (rapid amplification of cDNA ends) was carried out for amplification of the *NlPrx* full-length cDNA using the 5′ full RACE kit and 3′ full RACE core set (Takara Bio, Takara, Japan) according to the manufacturer’s instructions. Template was generated from a mixture of RNA from GX-P, R-P, and S-P. The specific primers used for amplification of *NlPrx* cDNA are detailed in S6 Table. PCR products were cloned into the pMD-18T vector (Takara Bio) and sequenced by IGE Biotechnology (Guangzhou, China). The full-length open reading frame sequence of *NlPrx* was assembled from all sequencing results and verified by PCR using the primers detailed in S6 Table. The predicted protein sequence was analyzed using the Conserved Domain Database search service CD-Search from the NCBI database.

### *NlPrx* promoter activity assay

Three stepwise deletion fragments of the *NlPrx* promoter region were PCR-amplified from GX-P-HR genomic DNA samples. The fragments ranged from +193 to −288 (not containing the sweep allele T65549), −373 (containing T65549), and −881 (containing T65549) (S6 Table). The resultant PCR products were purified by QIAquick PCR Purification Kit (Qiagen, Hilden, Germany), double cleaved with the restriction enzymes XhoI and KpnI, and cloned into the luciferase reporter plasmid pGL3-basic (Promega, Madison, WI, US), yielding the *NlPrx* promoter constructs P(−881/+193)-T65549, P(−373/+193)-T65549, and P(−288/+193). The obtained P(−881/+193)-T65549 and P(−373/+193)-T65549 constructs were sequenced to verify that they are the sweep allele containing T at the 65549 SNP site. Mutagenesis of the P(−881/+193)-T65549 construct was performed to make the counterpart allele construct, P(−881/+193)-65549A, by IGE Biotechnology (Guangzhou, China).

*T*. *ni*(Hi5) cells were transfected with a mixture of 0.1 ng of internal control plasmid (pRL-CMV vector, Promega), 0.6 μl of Fugent HP (Promega), and 0.2 μg of construct in Grace’s medium without FBS [41]. After 48 h, the cells were lysed in 1× passive lysis buffer (Promega). Finally, the luciferase activities were measured using the Dual-Luciferase Reporter Assay System (Promega). All transfections were independently repeated 3 times.

### qRT-PCR analyses

Total RNA was extracted from *N*. *lugens* samples using the Total RNA Kit (Omega, Norcross, GA, US). One microgram of total RNA was reverse transcribed to cDNA in a 20-μl reaction system using PrimeScript Reverse Transcriptase (Takara, Kyoto, Japan). All primers for the qRT-PCR analysis of *NlPrx*, *CYP6ER1*, and *CYP6AY1* genes are listed in S6 Table. The qRT-PCR was performed on a LightCycler 480 system (Roche, Indianapolis, IN, US) using SYBR Premix Ex Taq Kit (Roche) according to the manufacturer’s protocol. The housekeeping gene *β-actin* was used as endogenous control [84]. Three biological replicates of 3 technical replicates each were performed for each control or treatment, and relative expression levels were calculated with the ΔCt method and reported as 2^−Δ*C*T^.

### Time courses of *NlPrx* expression and ROS production and survival in the presence of imidacloprid

Due to the limited availability of 1-day-old *N*. *lugens* female adults, the time course experiment was done on 3 consecutive days. On day 1, 3 replicates of 50 1-day-old *N*. *lugens* female adults each from S-P and R-P were treated with solvent control or 10 mg/l imidacloprid using the rice stem dipping method described above. Ten live insects were randomly taken out from each replicate at 0 (before treatment), 12, and 24 h after treatment and used for measurement of ROS (5 insects) and *NlPrx* expression (5 insects). The 5 insects for ROS measurement were ground in phosphate buffered saline (PBS, pH 7.4) and incubated in PBS containing 10 μM DCFH-DA (Invitrogen) for 1 h in a dark room [85]. Then, the fluorescence values of each replicate of each population were recorded using a microplate reader (Tristar LB941, Berthold Technologies, Bad Wildbad, Germany) with 485-nm excitation and 535-nm emission, and adjusted with the blank control of the same volume of PBS and DCFH-DA. The values were further normalized to protein contents.

Similarly, the same replicates and numbers of 1-day-old female adults from each population were exposed to the solvent control or imidacloprid on day 2 and day 3. Ten live insects were randomly withdrawn from each replicate of the day 2 insects at 36 h for measurement of ROS and *NlPrx* expression. For the day 3 insects, we observed and recorded the numbers of live and dead insects at 0, 12, 24, 36, and 48 h, and randomly drew 10 live insects from each replicate at 48 h for measurement of ROS and *NlPrx* expression.

Furthermore, the same experiment as described above was conducted on the 1-day-old *N*. *lugens* female adults from GD-P-2020 to validate the field relevance of the observed phenomenon.

### Establishment and comparison of the R-P_TT and R-P_AA isogenic lines

Two isogenic lines, one with the homozygous sweep allele T65549 (named R-P_TT) and the other with the homozygous counterpart allele 65549A (named R-P_AA), were generated from the highly resistant R-P strain as diagramed in Fig 4A. Briefly, each newly emerged female adult was randomly paired with a male adult in a plastic cage with fresh rice plants. After laying eggs for 7 days, the parent pairs were individually genotyped. The offspring whose parents both carried the homozygous T65549 allele of *NlPrx* were pooled together to establish the R-P_TT line, whereas the offspring whose parents both had the homozygous 65549A allele of *NlPrx* were pooled together to yield the R-P_AA line.

Under normal rearing conditions, we measured the ROS level and life history traits of the 2 isogenic lines. Specifically, ROS level was measured using 1-day-old female adults as described above. For measurement of life history traits, multiple mated female adults were randomly chosen from each of the 2 isogenic lines and kept in 2 separated cages to lay eggs on fresh rice plants. When the eggs started to hatch, we transferred the newly hatching nymphs of each isogenic line to more than 10 vials (20 nymphs/vial) with fresh rice plants to record their growth, development, and longevity. When the adults emerged, we randomly paired 10 virgin females and 10 virgin males from the 10 vials and introduced each of the 10 pairs of each isogenic line into a plastic cage with fresh rice plants for oviposition. The total number of offspring of each pair was counted and recorded after 15 days. At least 6 of the 10 pairs successfully mated and produced offspring for both isogenic lines.

We also exposed 1-day-old female adults of the 2 isogenic lines to solvent control, 10 mg/l imidacloprid alone, or combination 10 mg/l imidacloprid + 5 mg/l P450 inhibitor PBO. For the combination treatment, we pretreated the 2 isogenic lines on rice stems dipped with 5 mg/l PBO for 5 h, and then transferred the insects onto rice stems dipped with 10 mg/l imidacloprid + 5 mg/l PBO for 19 h. There were 2 additional combination treatments for the R-P_TT line: 10 mg/l imidacloprid + 0.1 mmol peroxiredoxin II inhibitor *Conoidin A* and 10 mg/l imidacloprid + 0.1 mmol *Conoidin A* + 5 mg/l PBO. For each treatment and control, 3 replicates of 20 1-day-old female adults were treated. Survival and ROS level of the survivors were checked or measured 24 h post-exposure.

### RNAi silencing of *NlPrx* in R-P

The full coding sequence of *NlPrx* was cloned into the pMD-18T vector (Takara Bio) and sequenced by IGE Biotechnology before dsRNA synthesis. The sequence-verified plasmid was used to amplify a 355-bp template for production of the dsRNA of *NlPrx* (ds*NlPrx*). The fragment of the green fluorescent protein (GFP) gene (ACY56286) was amplified with the primers reported by Chen et al. [84] (S6 Table) and used as the template for production of the negative control dsRNA. The dsRNAs of *NlPrx* (ds*NlPrx*) and *GFP* (ds*GFP*) were prepared using the T7 RiboMAX Express RNAi System (Promega, Madison, WI, US) following the manufacturer’s instructions.

One hundred nanoliters of the purified ds*NlPrx* or ds*GFP* (1 ng/nl) was injected into each of more than 200 2-day-old female adults of R-P according to the method of Zhai et al. [86]. The injected female adults were reared in a cage containing fresh rice plants at 26 ± 2 °C with 80% ± 10% humidity and a light/dark cycle of 16 h/8 h. At 12, 24, and 36 h post-injection, 3 replicates of 5 female adults each were randomly taken out from the cage, flash-frozen in liquid nitrogen, and stored at −80 °C for subsequent RNA extraction and qRT-PCR quantification of *NlPrx* transcripts using the method described above. After confirming the RNAi efficiency of ds*NlPrx*, injected female adults were randomly divided into 3 groups at 24 h post-injection. The 3 groups of female adults were transferred to the cages with rice stems dipped with 0 (solvent control), 10, or 50 mg/l imidacloprid solutions. Each group included 3 replicates of treatment. The ROS level of each replicate of the 3 groups was measured 24 h after insecticide treatment, and the mortality of each replicate was observed 48 h after treatment.

### Construction and contact bioassay of transgenic fly strains

The open reading frame of *NlPrx* cloned from the GX-P-HR sample was inserted into pValium20 vector to make the UAS-*NlPrx* construct. The UAS-*NlPrx* construct (1 ng/nl) was microinjected into the embryos (<30 min; 10 nl per embryo) of the y sc v nanos-integrase; attP2 *D*. *melanogaster* line using standard techniques. The resulting UAS-*NlPrx* transgenic line was crossed with the Act5C-GAL4 driver line for production of the GAL4/UAS-*NlPrx* progeny line that expresses the *NlPrx* gene.

Three pools of 10 adults each were used to exact 3 total RNA samples (3 replicates) from the UAS-*NlPrx* transgenic line, the Act5C-GAL4 driver line, and the GAL4/UAS-*NlPrx* progeny line, using the methods described above. RT-PCR gel analysis and qRT-PCR were conducted to detect and quantify the expression of *NlPrx* in the 3 *Drosophila* lines using the specific primers (S6 Table) for *NlPrx* and the reference housekeeping gene *DmActin* [87].

Every 20 1- to 3-day-old adults (10 males and 10 females) from the Act5C-GAL4 driver line, the UAS-*NlPrx* transgenic line, or the GAL4/UAS-*NlPrx* progeny line were placed in vials with 10 ml of corn meal medium containing 300 μg of imidacloprid or solvent control [88]. The ROS level was measured at 24 h after exposure to imidacloprid, and the mortality was calculated at 48 h after exposure to imidacloprid. The control and treatment were repeated 3 times for each line.

### Data analysis

All paired comparisons on allelic frequencies were analyzed using the *χ*^2^ test. The correlation between the *NlPrx* T65549 allele frequency and imidacloprid resistance of *N*. *lugens* was estimated using odds ratios and Fisher’s exact test as previously described [89], and was further established according to the linear regression analysis of T65549 allele frequencies and the Ln-transformation of resistance ratios of *N*. *lugens* populations in SPSS Statistics version 21 (IBM, US). The correlation between ROS level and the P450 *CYP6AY1* and *CYP6ER1* expression in GD-P-2020 was calculated according to the Pearson correlation in R. The paired comparisons on normalized mRNA levels, ROS levels, survival rates, and fitness-related traits were analyzed with the Student *t* test. All multiple comparisons on promoter activities, normalized mRNA levels, ROS levels, and survival rates were tested with 1-way ANOVA, followed by Duncan’s multiple range test (*P* < 0.05).

## Supporting information

S1 Data Excel spreadsheet with numerical raw data underlying Figs 1A–1C, 1E, 2A–2C, 3A–3E, 4B–4E, 5A–5F; S1 and S2 Figs (doi: 10.6084/m9.figshare.14177009). (XLSX)

S1 Fig The correlation analysis of SNP frequency between parallel samples of GX-P-HR and GX-P-LR in scaffold KN151899.1. Numerical values are provided in S1 Data (doi: 10.6084/m9.figshare.14177009).
(TIF)

S2 Fig The *F*_ST_ distribution spanning scaffold KN155291.1. Numerical values are provided in S1 Data (doi: 10.6084/m9.figshare.14177009).
(TIF)

S3 Fig Nucleotide and amino acid sequences of *N*. *lugens* peroxiredoxin. The start and stop codons are shaded gray. The catalytic triad sites and peroxidatic sites are boxed.
(TIF)

S4 Fig RT-PCR gel detection of *NlPrx* transcripts in the GAL4/UAS-*NlPrx* progeny line (lanes 1, 2, and 3; 3 biological replicates), UAS-*NlPrx* transgenic line (lanes 4, 5, and 6; 3 biological replicates), and Act5C-GAL4 driver line (lanes 7, 8, and 9; 3 biological replicates) of *D*. *melanogaster*. Original images for gels are provided in S1 Raw Images.
(TIF)

S1 Raw Images Original images for gel results of S4 Fig. (TIF)

S1 Table Resistance levels of 6 *N*. *lugens* populations/subpopulations to imidacloprid. (DOCX)

S2 Table Discrimination of the high-resistant (GX-P-HR) and low-resistant (GX-P-LR) individuals from the imidacloprid-resistant GX-P population of *N*. *lugens*. (DOCX)

S3 Table Summary of the genomic resequencing data of GX-P-HR and GX-P-LR individuals. (DOCX)

S4 Table One hundred thirty-nine genomic regions with signal of sweep Z(*F*_ST_) > 4. (XLSX)

S5 Table GO functional enrichment of candidate genes. (XLSX)

S6 Table The primers used in this study. (DOCX)

## Abbreviations

alt: alternative
Bt: *Bacillus thuringiensis*
ds: double-stranded
GO: Gene Ontology
LC_50_: 50% lethal concentration
PBO: piperonyl butoxide
qRT-PCR: quantitative reverse transcription PCR
ref: reference
RNAi: RNA interference
ROS: reactive oxygen species
RT-PCR: reverse transcription PCR
