# Supplementary figures and images for: Peroxiredoxin alleviates the fitness costs of imidacloprid resistance in an insect pest of rice
Source: PLoS Biol. 2021 Apr 12;19(4):e3001190. doi: 10.1371/journal.pbio.3001190 (PMC8062100; doi:10.1371/journal.pbio.3001190)

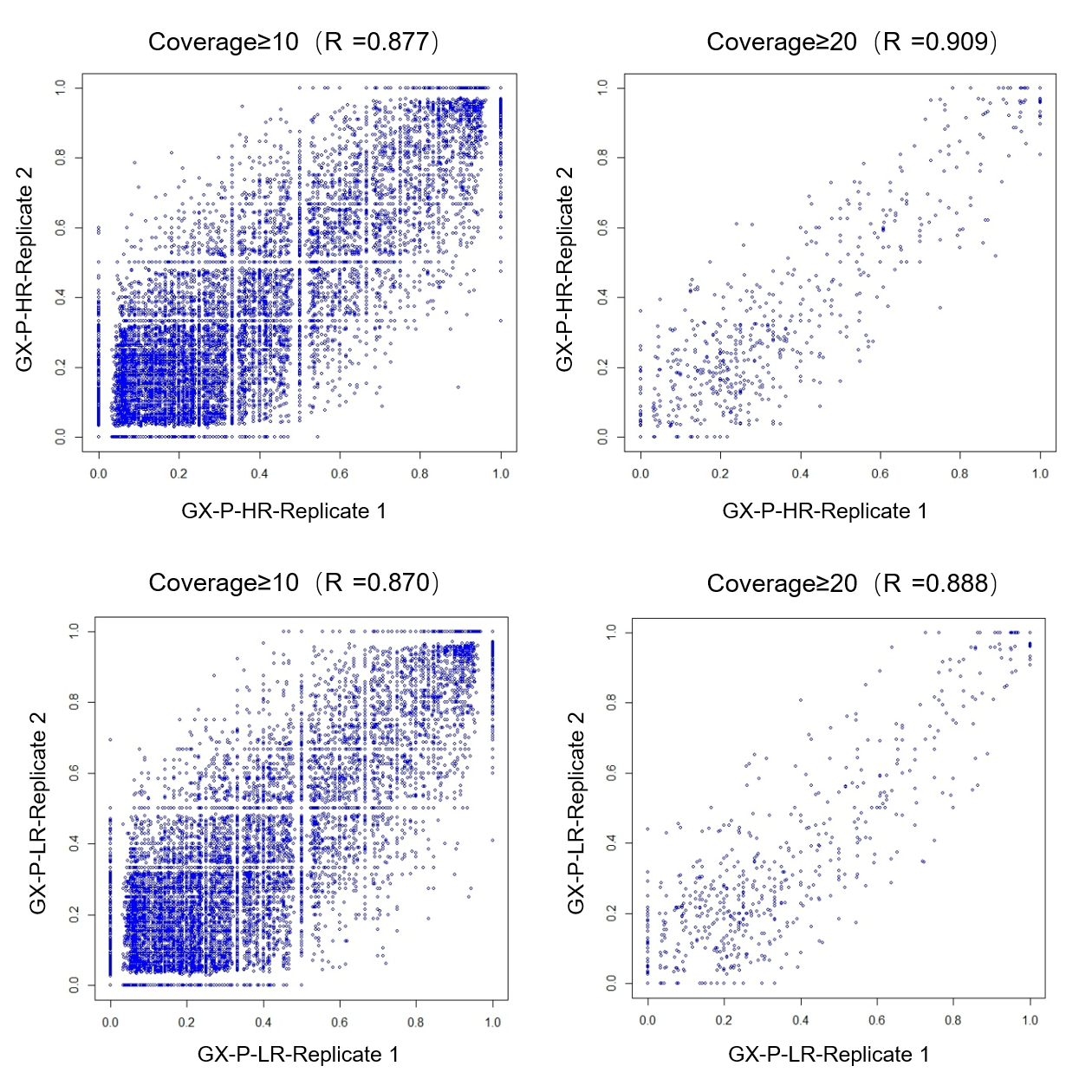

Supplement: S1 Fig — Numerical values are provided in S1 Data (doi: 10.6084/m9.figshare.14177009). (TIF) [file pbio.3001190.s002.tif]

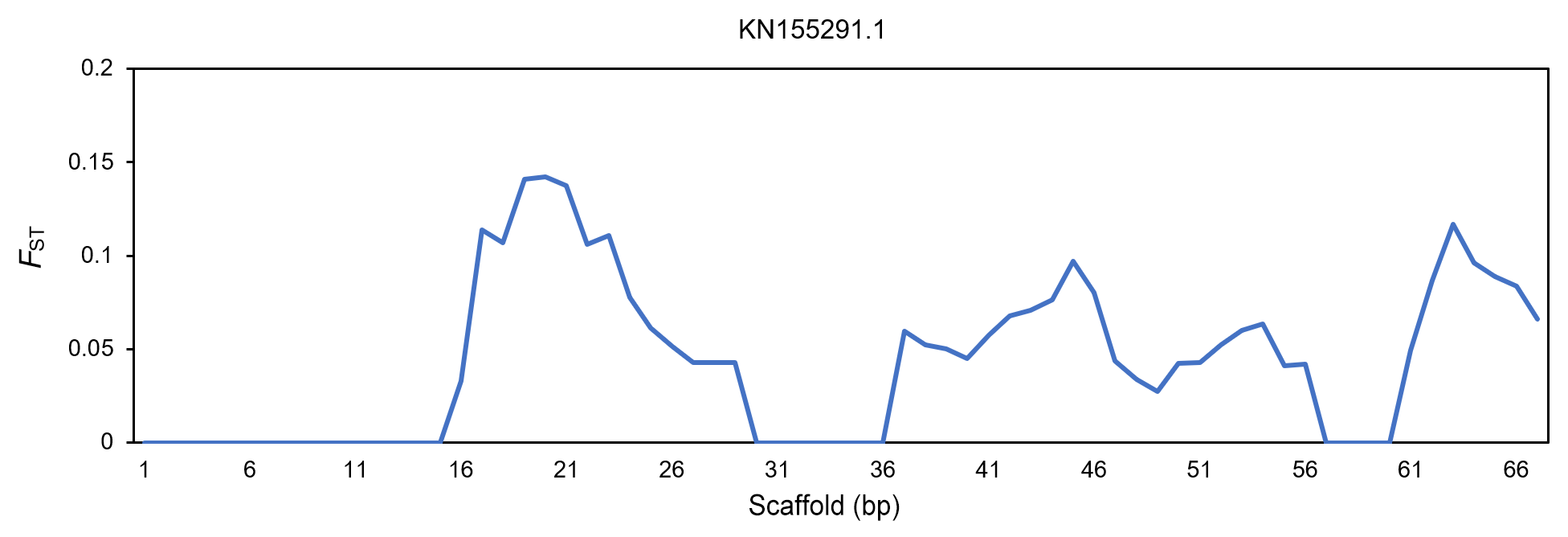

Supplement: S2 Fig — Numerical values are provided in S1 Data (doi: 10.6084/m9.figshare.14177009). (TIF) [file pbio.3001190.s003.tif]

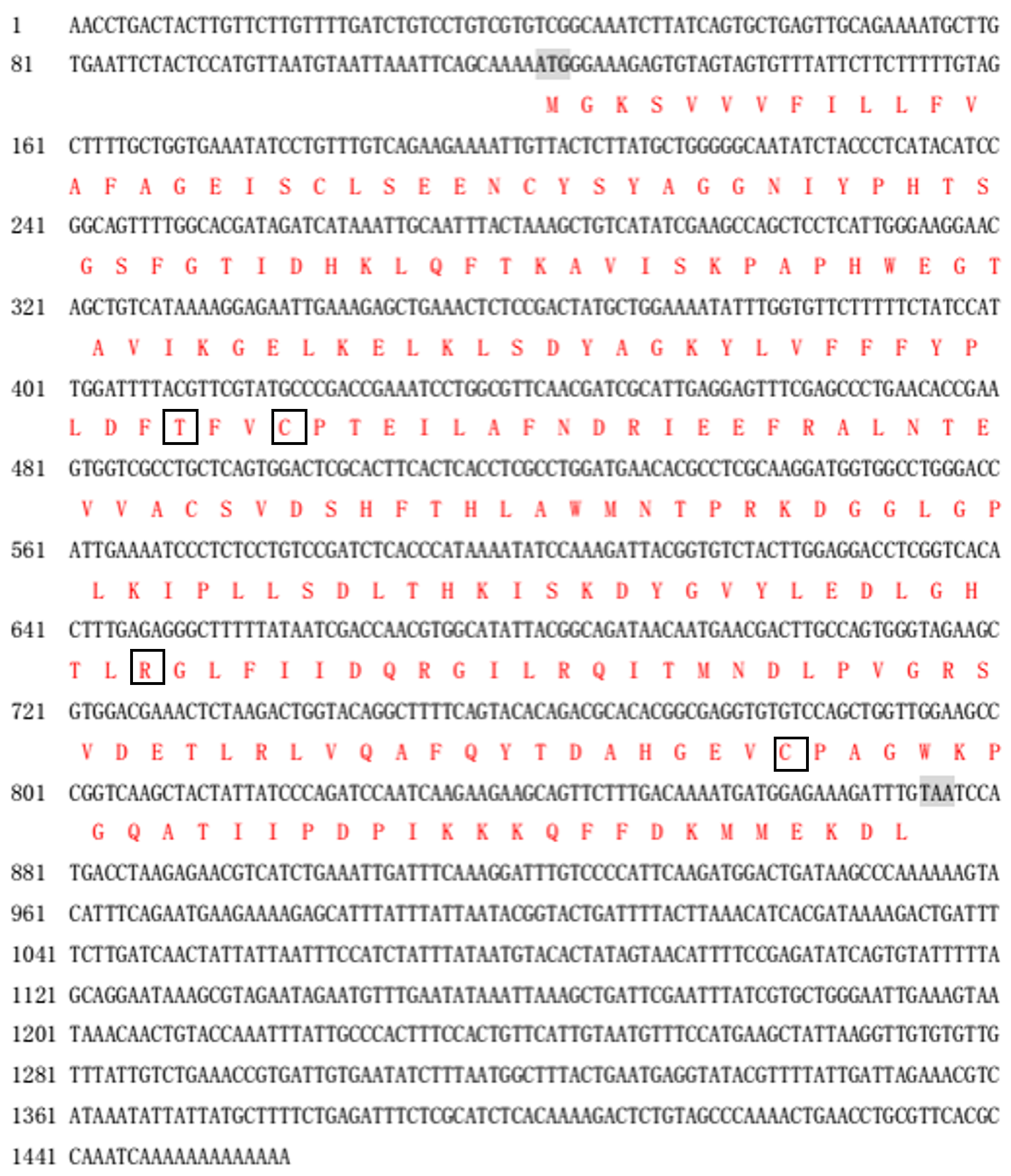

Supplement: S3 Fig — The start and stop codons are shaded gray. The catalytic triad sites and peroxidatic sites are boxed. (TIF) [file pbio.3001190.s004.tif]

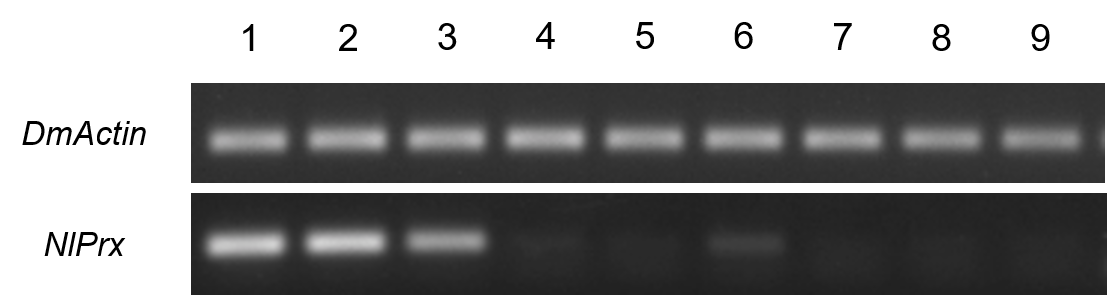

Supplement: S4 Fig — Original images for gels are provided in S1 Raw Images. (TIF) [file pbio.3001190.s005.tif]

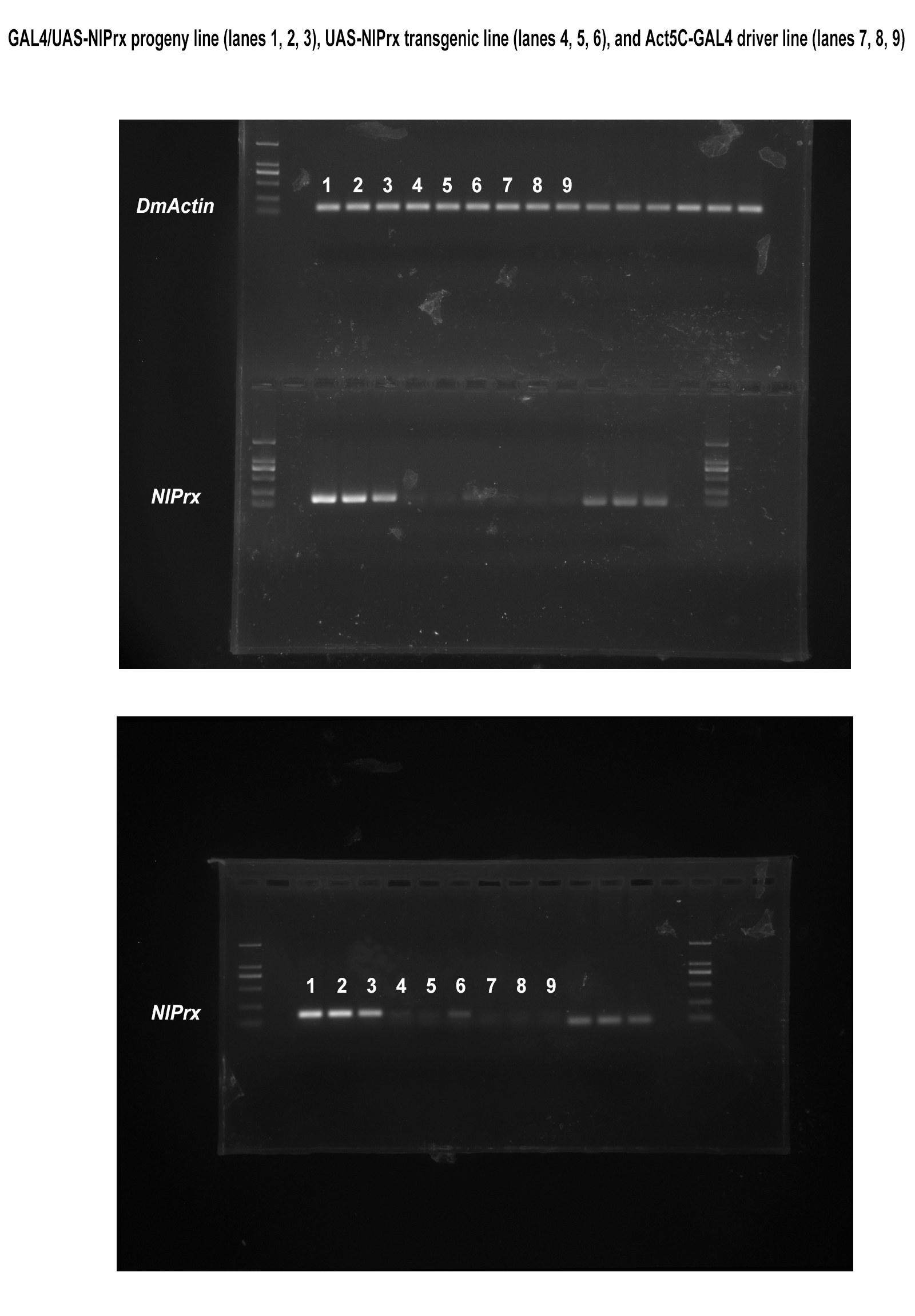

Supplement: S1 Raw Images — (TIF) [file pbio.3001190.s006.tif]
