# Supplementary material for: Peroxiredoxin alleviates the fitness costs of imidacloprid resistance in an insect pest of rice
Source: PLoS Biol. 2021 Apr 12;19(4):e3001190. doi: 10.1371/journal.pbio.3001190 (PMC8062100; doi:10.1371/journal.pbio.3001190)
Supplement: S1 Table — (DOCX) [file pbio.3001190.s007.docx]

**S1 Table. Resistance levels of 6 *N. lugens* populations to imidacloprid.**

| Populations | LC-p line (y =) | LC_50_ (95% CL) mg ai/L | Resistance ratio^a^ |
| --- | --- | --- | --- |
| GX-P | 3.1566 + 1.2363x | 30.978 (23.409-43.380) | 110.64 |
| GX-P-HR | N/A | >300 | >1071.43 |
| GX-P-LR | N/A | <5 | <17.857 |
| GD-P-2014-F0 | 3.6384+0.7520x | 64.6468 (38.4482-147.3771) | 230.88 |
| S-P | 4.3985 + 1.5212x | 2.485 (1.381-3.581) | 8.875 |
| R-P | 2.3973 + 1.5166x | 52.014 (44.499-61.426) | 185.76 |
| GD-P-2020 | 2.5056 + 1.179x | 130.5 (93.415-210.91) | 466.07 |
| S strain in Wu et al. (2018) | N/A | 0.28 (0.23–0.35) | 1 |
| S strain in Wang et al. (2008, 2009) | N/A | 0.08 (0.05-0.11) | N/A |

LC, lethal concentration; CL, confidence limit; N/A, data not available.

a: The presented resistance ratios are based on LC_50_ value of the S strain in Wu et al. (2018).
