# Supplementary material for: Peroxiredoxin alleviates the fitness costs of imidacloprid resistance in an insect pest of rice
Source: PLoS Biol. 2021 Apr 12;19(4):e3001190. doi: 10.1371/journal.pbio.3001190 (PMC8062100; doi:10.1371/journal.pbio.3001190)
Supplement: S2 Table — (DOCX) [file pbio.3001190.s008.docx]

**S2 Table. Discrimination of the high resistant (GX-P-HR) and low resistant (GX-P-LR) individuals from the imidacloprid-resistant GX-P population of *N. lugens.***

| Doses (mg/L) | Test dates | No. of tested insects | No. of dead insects | | | | | No. of survivors | Mortality (%) |
| --- | --- | --- | --- | --- | --- | --- | --- | --- | --- |
|  |  |  | Day 1 | Day 2 | Day 3 | Day 4 | Total |  |  |
| 5 | 08-25-2011 | 1650 | 31 | **37^a^** | **33^a^** | 25 | 126 | 1524 | 7.64 |
|  | 08-26-2011 | 1050 | 23 | **19^b^** | **19^b^** | 14 | 75 | 975 | 7.14 |
| 300 | 08-25-2011 | 3150 | ND | ND | ND | ND | 2771 | **379^c^** | 87.97 |

a: The 70 individuals died on Day 2-3 post exposure to the LC_10_ dose (5 mg/L) of imidacloprid are considered as the low resistant or near susceptible individuals (GX-P-LR). We randomly selected 30 from the 70 just dead insects and pooled them together as one replicate of GX-P-LR for subsequent genomic DNA extraction and high throughput sequencing.

b: Likewise, we randomly selected 30 from the 38 just dead insects on Day 2-3 and pooled them together as another replicate of GX-P-LR for subsequent genomic DNA extraction and high throughput sequencing.

c: The 379 insects survived from the LC_90_ dose (300 mg/L) treatment are considered as the highly resistant individuals (GX-P-HR). Two replicates of 30 each were randomly selected from the 379 survivors and used for subsequent genomic DNA extraction and high throughput sequencing.

ND: no data. Unlike the discrimination of the GX-P-LR individuals, we did not check and record dead insects daily for selection of GX-P-HR individuals.
