# Supplementary material for: Peroxiredoxin alleviates the fitness costs of imidacloprid resistance in an insect pest of rice
Source: PLoS Biol. 2021 Apr 12;19(4):e3001190. doi: 10.1371/journal.pbio.3001190 (PMC8062100; doi:10.1371/journal.pbio.3001190)
Supplement: S3 Table — (DOCX) [file pbio.3001190.s009.docx]

**S3 Table. Summary of the genomic resequencing data of GX-P-HR and GX-P-LR individuals.**

| Sample | GX-P-R-1 | GX-P-R-2 | GX-P-S-1 | GX-P-S-2 |
| --- | --- | --- | --- | --- |
| Number of clean reads | 188,184,006 | 236,177,420 | 219,608,142 | 214,158,802 |
| Sequencing depth (×) | 17.108 | 21.471 | 19.964 | 19.469 |
| Mapped reads | 142,134,102 | 175,985,883 | 162.794.486 | 156,325,957 |
| Mapping rate (%) | 75.53 | 74.51 | 74.13 | 73.00 |
| Genome coverage (%) | 94.6 | 95.6 | 95.2 | 95.2 |
