# Supplementary material for: Peroxiredoxin alleviates the fitness costs of imidacloprid resistance in an insect pest of rice
Source: PLoS Biol. 2021 Apr 12;19(4):e3001190. doi: 10.1371/journal.pbio.3001190 (PMC8062100; doi:10.1371/journal.pbio.3001190)
Supplement: S6 Table — (DOCX) [file pbio.3001190.s012.docx]

**S6 Table. The primers used in this study.**

| **Primer name** | **Sequence** | **Purpose** |
| --- | --- | --- |
| KN155291-F1 | ATACAGACCAATACCCAAAAACCAC | Genotyping for site 15914 |
| KN155291-R1 | TCAAACCAGCCAAGTAGGAAC | Genotyping for site 15914 |
| KN155291-F2 | GTCGCCTGTTGGTGATGGAAG | Genotyping for site 20089 |
| KN155291-R2 | AAAATATCGGGGACCGAGCTT | Genotyping for site 20089 |
| KN155291-F3 | GATAAGAtttgccgacacgacagg | Genotyping for site 65549 |
| KN155291-R3 | GTGTTCACTGCCGTAACTTTGTCT | Genotyping for site 65549 |
| NlPrx-Pro-F | gcgcggtaccTATATATATATGATATTACATTTAG | *NlPrx* promoter-pGL3 plasmids construction |
| NlPrx-Pro-R | gcgcctcgagCTGACAAACAGGATATTTCACCAGCA | *NlPrx* promoter-pGL3 plasmids construction |
| NlPrx-Pro-D1-F | gcgcggtaccCTGATATCAATGACTTACTTTGTTATCC | *NlPrx* promoter deletion 1-pGL3 plasmids construction |
| NlPrx-Pro-D2-F | gcgcggtaccGCCTAAATCAAAAATTCCAATGACTG | *NlPrx* promoter deletion 2-pGL3 plasmids construction |
| NlPrx-F | ATCCCTCTCCTGTCCGATCTC | Quatitative real-time PCR for verification of *NlPrx* |
| NlPrx-R | GTTTCGTCCACGCTTCTACCC | Quatitative real-time PCR for verification of *NlPrx* |
| CYP6ER1_F | ATTCCGGTCTATGCGCTTC | Quatitative real-time PCR (amplification of P450 gene *CYP6ER1*) |
| CYP6ER1_R | TGGATTGGCGCTCTCTTACT | Quatitative real-time PCR (amplification of P450 gene *CYP6ER1*) |
| CYP6AY1_F | GCTTTTGCTTGTATGAGTTGGC | Quatitative real-time PCR (amplification of P450 gene *CYP6AY1*) |
| CYP6AY1_R | AGGTGGGTATTTCCTCATCGT | Quatitative real-time PCR (amplification of P450 gene *CYP6AY1*) |
| Actin_F | TGCGTGACATCAAGGAGA | Quatitative real-time PCR (amplification of housekeeping gene) |
| Actin_R | GGAAGGAAGGCTGGAACA | Quatitative real-time PCR (amplification of housekeeping gene) |
| NlPrx-ds-F | TAGCTTTTGCTGGTGAAATATCCTG | synthesis of dsRNA for *NlPrx* |
| NlPrx-ds-R | GTGAAGTGCGAGTCCACTGAG | synthesis of dsRNA for *NlPrx* |
| NlPrx-ds-FT7 | GGATCCTAATACGACTCACTATAGGTAGCTTTTGCTGGTGAAATATCCTG | synthesis of dsRNA for *NlPrx* |
| NlPrx-ds-RT7 | GGATCCTAATACGACTCACTATAGGGTGAAGTGCGAGTCCACTGAG | synthesis of dsRNA for *NlPrx* |
| GFP-ds-F | AAGGGCGAGGAGCTGTTCACCG | synthesis of dsRNA for *GFP* |
| GFP-ds-R | CAGCAGGACCATGTGATCGCGC | synthesis of dsRNA for *GFP* |
| GFP-ds-FT7 | GGATCCTAATACGACTCACTATAGGAAGGGCGAGGAGCTGTTCACCG | synthesis of dsRNA for *GFP* |
| GFP-ds-RT7 | GGATCCTAATACGACTCACTATAGGCAGCAGGACCATGTGATCGCGC | synthesis of dsRNA for *GFP* |
| NlPrx-ORF-F | ATGGGAAAGAGTGTAGTAGTGTTT | Amplification of full-length coding sequence |
| NlPrx-ORF-R | TTACAAATCTTTCTCCATCATTTT | Amplification of full-length coding sequence |
| NlPrx -5'-GSP1 | GAGGTGAGTGAAGTGCGAGTCC | 5' RACE |
| NlPrx -5'-GSP2 | AATGAGGAGCTGGCTTCGATAT | 5' RACE |
| NlPrx -3'-GSP1 | AAAGATTACGGTGTCTACTTGGAG | 3' RACE |
| NlPrx -3'-GSP2 | ATAACAATGAACGACTTGCCAG | 3' RACE |
|  |  |  |
| NlPrx-ORF-F’ | GCTCTAGAATGGGAAAGAGTGTAGTAGTGTTT | UAS-NlPrx plasmid construction |
| NlPrx-ORF-R’ | CGGAATTCTTACAAATCTTTCTCCATCATTTT | UAS-NlPrx plasmid construction |
| NlPrx-RT-F | ATCCCTCTCCTGTCCGATCTC | RT-PCR detection of *NlPrx* transcripts in Drosophila lines |
| NlPrx-RT-R | GTTTCGTCCACGCTTCTACCC | RT-PCR detection of *NlPrx* transcripts in *Drosophila* lines |
| DmActin-F | GCGTCGGTCAATTCAATCTT | RT-PCR detection of *NlPrx* transcripts in *Drosophila* lines |
| DmActin-R | AAGCTGCAACCTCTTCGTCA | RT-PCR detection of *NlPrx* transcripts in *Drosophila* lines |
